# scRNA sequencing uncovers a TCF4-dependent transcription factor network regulating commissure development in mouse

**DOI:** 10.1242/dev.196022

**Published:** 2021-07-19

**Authors:** Marie-Theres Wittmann, Sayako Katada, Elisabeth Sock, Philipp Kirchner, Arif B. Ekici, Michael Wegner, Kinichi Nakashima, Dieter Chichung Lie, André Reis

**Affiliations:** 1 Institute of Human Genetics, Universitätsklinikum Erlangen, Friedrich-Alexander-Universität Erlangen-Nürnberg (FAU), 91054 Erlangen, Germany; 2 Institute of Biochemistry, Emil Fischer Center, Friedrich-Alexander-Universität Erlangen-Nürnberg, 91054 Erlangen, Germany; 3 Department of Stem Cell Biology and Medicine, Graduate School of Medical Sciences, Kyushu University, Fukuoka 812-8582, Japan

**Keywords:** TCF4, Single-cell RNA sequencing, Gene regulatory networks, Protein-protein interaction, SOX11, Commissure development, Mouse

## Abstract

Transcription factor 4 (TCF4) is a crucial regulator of neurodevelopment and has been linked to the pathogenesis of autism, intellectual disability and schizophrenia. As a class I bHLH transcription factor (TF), it is assumed that TCF4 exerts its neurodevelopmental functions through dimerization with proneural class II bHLH TFs. Here, we aim to identify TF partners of TCF4 in the control of interhemispheric connectivity formation. Using a new bioinformatic strategy integrating TF expression levels and regulon activities from single cell RNA-sequencing data, we find evidence that TCF4 interacts with non-bHLH TFs and modulates their transcriptional activity in *Satb2*^+^ intercortical projection neurons. Notably, this network comprises regulators linked to the pathogenesis of neurodevelopmental disorders, e.g. *FOXG1*, *SOX11* and *BRG1*. In support of the functional interaction of TCF4 with non-bHLH TFs, we find that TCF4 and SOX11 biochemically interact and cooperatively control commissure formation *in vivo*, and regulate the transcription of genes implicated in this process. In addition to identifying new candidate interactors of TCF4 in neurodevelopment, this study illustrates how scRNA-Seq data can be leveraged to predict TF networks in neurodevelopmental processes.

## Abstract (precis)

**Summary:** Single-cell RNA sequencing identifies interactions of TCF4 with non-bHLH transcription factors linked to neurodevelopmental and neuropsychiatric disease in the regulation of interhemispheric projection neuron development.

## INTRODUCTION

Transcription factor 4 (TCF4) – not to be confused with the canonical Wnt signaling-associated transcriptional regulator T-cell factor 4 (transcription factor 7-like 2, TCF7L2) – is a crucial transcriptional regulator in forebrain development. Variants in *TCF4* have been associated with schizophrenia, autism and intellectual disability and *TCF4* haploinsufficiency causes the neurodevelopmental disorder Pitt–Hopkins syndrome (PTHS) (OMIM 610954) (Stefansson et al., 2009; Steinberg et al., 2011; De Rubeis et al., 2014; Zweier et al., 2007; Amiel et al., 2007).

Analyses of transgenic mice have shown that alterations of TCF4 dosage cause disruptions in neocortical neuronal migration, specification of neuronal subtypes, dendrite and synapse formation (Li et al., 2019; Page et al., 2018). Moreover, homozygous *Tcf4* loss of function results in deletion of all forebrain commissures in mice (Mesman et al., 2020), whereas *TCF4* haploinsufficiency in humans and in mice is associated with callosal dysgenesis (Jung et al., 2018; Zweier et al., 2007), indicating that *TCF4* is part of a conserved genetic network controlling the formation of callosal connections.

TCF4 is a class I basic helix-loop-helix (bHLH) transcription factor (TF) and its transcriptional output is highly dependent on its interaction partners (Le Dréau et al., 2018). Traditionally, it is assumed that TCF4 executes its function through homodimerization or heterodimerization with proneural class II bHLH TFs, such as the neurogenin or NeuroD family (Bertrand et al., 2002). Recent *in vitro* analysis of murine neural precursor cells raised the intriguing possibility that TCF4 has the potential to form ‘non-canonical’ interactions, i.e. to interact with transcriptional regulators outside of the bHLH class (Moen et al., 2017). *In vivo*, cell type-specific interactors of TCF4 have not been established and it remains to be determined whether interactions of TCF4 with non-bHLH TFs are involved in the regulation of specific neurodevelopmental processes.

Here, we focused on TCF4-dependent transcriptional networks in mouse intercortical projection neurons. First, we corroborated the recent finding that loss of *Tcf4* results in the complete agenesis of forebrain commissures in mice (Mesman et al., 2020). In subsequent analyses, we used single-cell RNA sequencing (scRNA-Seq) to uncover TCF4-dependent transcriptional networks in *Satb2*-expressing intercortical projection neurons. Integration of TF expression levels and regulon activities surprisingly predicted that TCF4 engages in interaction with numerous TFs outside of the bHLH class and regulates their transcriptional activity. Similar to TCF4, these interactors are often associated with neurodevelopmental disorders, such as intellectual disability and autism. Reinforcing our model of TCF4-dependent transcriptional regulation by interaction, *in vitro* and *in vivo* analyses of the interaction between TCF4 and the regulator SOX11 revealed a synergistic effect of these TFs on anterior commissure (AC) and corpus callosum (CC) formation. Collectively, our study provides insight into TCF4-dependent interactions in the control of commissure formation and proposes a new strategy to harness scRNA-Seq data to predict *in vivo* transcription factor interactions in a cell type-specific manner.

## RESULTS

### *Tcf4* knockout abolishes commissure development

**Fig. 1.**
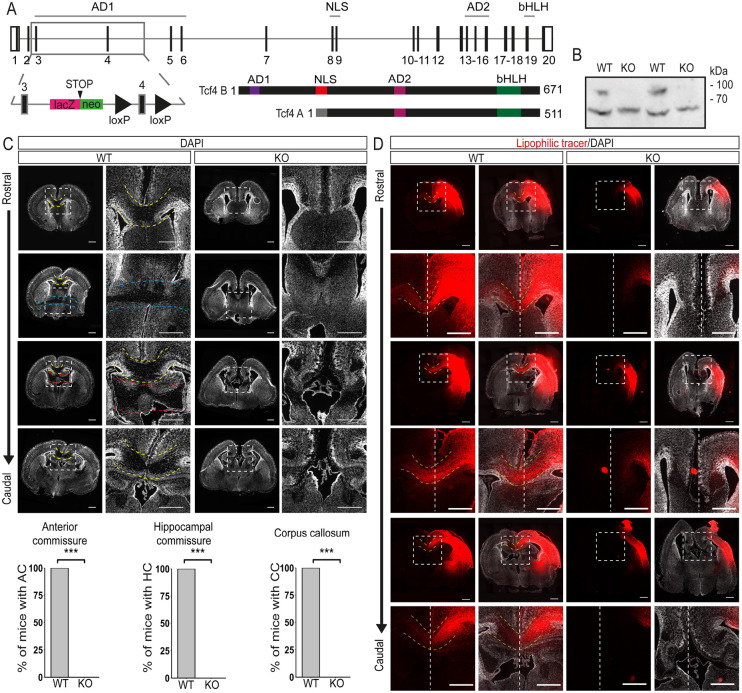
**Loss of *Tcf4* disrupts commissure formation.** (A) Schematic of the *Tcf4* gene, the ‘knockout-first’ conditional allele and the two main TCF4 isoforms. (B) Western blot analysis of neocortical extracts from E18.5 WT or *Tcf4*KO mice using anti-TCF4 antibody. The blot presented is cropped. The longest isoform of TCF4 is missing in the KO samples (*n*=3). (C) Representative overview and magnification images of DAPI-stained brain sections at P0 showing the loss of the three commissure systems in *Tcf4*KO mice. Images on the right are magnifications of the boxed areas. Yellow dashed lines indicate the CC crossing the midline. Blue dashed lines indicate the AC and red dashed lines the hippocampal commissure (HC). Quantification of animals showing each commissural system is presented below (*n*=8, mean±s.d). ***P≤0.001. (D) Representative overview and magnification images of lipophilic tracer (red)-treated brains at P0 with or without DAPI staining (white). Images at the bottom are magnifications of the boxed areas. White dashed lines indicate the midline and yellow dashed lines the CC. Note that lipophilic tracer signal can be detected in the contralateral hemispheres only in WT animals (*n*=3). Scale bars: 500 µm. For more overview and magnification images, see Figs S1 and S2.

Mesman and colleagues (Mesman et al., 2020) recently reported that a homozygous mutation removing the DNA-binding bHLH domain from all TCF4 isoforms is associated with loss of the forebrain commissural system. Here, we analyzed *Tcf4* knockout mice (*Tcf4*KO) generated by the homozygous insertion of a *lacZ*/neomycin cassette before exon 4 of *Tcf4* (Fig. 1A). Effectiveness of the *Tcf4* knockout was assessed by western blot as there are *Tcf4* isoforms with transcriptional start sites after exon 4 (Sepp et al., 2011). Our analysis confirmed the loss of the longest TCF4 isoform (TCF4B) in the knockout; expression of a shorter isoform (TCF4A) persisted (Fig. 1B). Despite the residual TCF4 expression, *Tcf4*KO mice died shortly after birth, indicating the importance of the long TCF4 isoform during development.

**Fig. 2.**
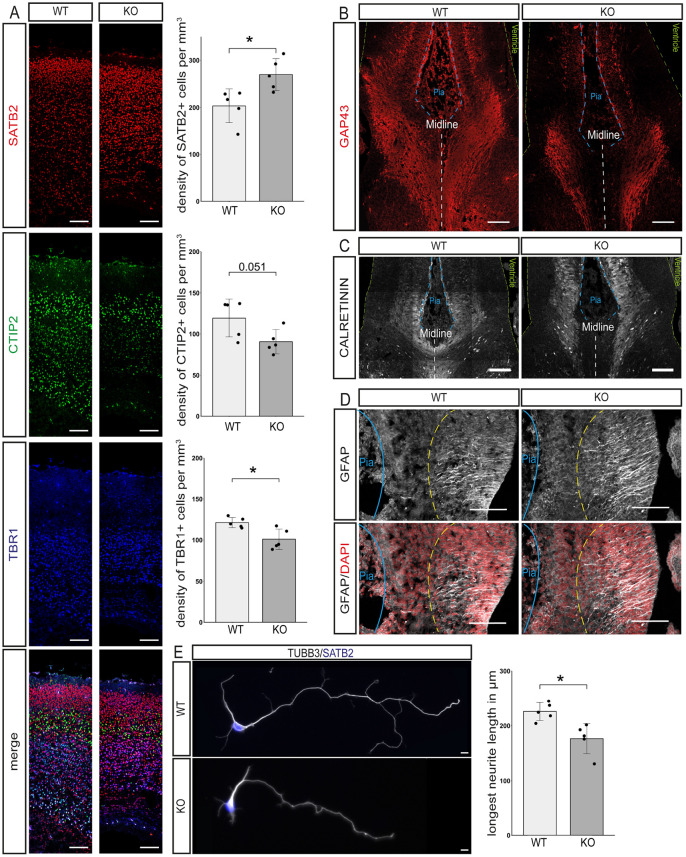
**Loss of *Tcf4* disrupts cortical layering and neurite outgrowth.** (A) Representative images of the neuronal markers SATB2 (upper cortical layers, red), CTIP2 (layer V, green) and TBR1 (layer VI, blue) and quantification of the density of cells expressing these markers in mm^3^. A significant increase in SATB2^+^ neurons is observable, as is a significant decrease in TBR1^+^ cells (*n*=5, mean±s.d.; *P*-values were determined with two-tailed Student's *t*-tests; SATB2^+^ cells per mm^3^: WT 203±32; KO 270±30; *P*=0.017; CTIP2^+^ per mm^3^: WT 119±21; KO 91±13; *P*=0.051; TBR1^+^ cells per mm^3^: WT 121±5; KO 101±11; *P*=0.018). Scale bars: 100 µm. (B) Representative images of GAP43 immunostaining at the midline of E16.5 mouse brains. Green dashed lines represent the ventricular surface, blue dashed lines the pial surface and white dashed lines the midline (*n*=3). Scale bars: 100 µm. (C) Representative images of calretinin expression at the midline at E16.5. Green dashed lines represent the ventricular surface, blue dashed lines the pial surface and white dashed lines represent the midline (*n*=3). Scale bars: 100 µm. (D) Representative images of GFAP staining at E16.5. Blue lines represent the pial surface. Yellow dashed lines represent the end of the processes of the glial wedge glia (*n*=3). Scale bars: 100 µm. (E) Representative images of primary cortical neurons stained for TUBB3 (gray) and SATB2 (blue) and the quantification of the length of the longest neurite. *Tcf4*KO neurons show a reduced neurite length (*n*=5; 20 neurons per animal; longest neurite length in µm: WT 226.02±14.76; KO 176.59±24.45; *P*=0.012). Scale bars: 10 µm.

In accordance with the findings of Mesman and colleagues  (Mesman et al., 2020), postnatal day (P) 0 TCF4 KO mice showed complete absence of the forebrain commissure system, i.e. CC, AC and hippocampal commissure (Fig. 1C,D, Fig. S1). Staining for the upper layer and interhemispheric projection neuron (IPN) marker SATB2 (Alcamo et al., 2008; Britanova et al., 2008) revealed a significant increase in SATB2-positive neurons in knockout (KO) compared with wild-type (WT) forebrains (SATB2^+^ cells per mm^3^: WT 203±32; KO 270±30; *P*=0.017; Fig. 2A), which appeared to be generated at the expense of TBR1-positive (TBR1^+^ cells per mm^3^: WT 121±5; KO 101±11; *P*=0.018; Fig. 2A) but not of CTIP2 (BCL11B)-positive deep layer neurons (CTIP2^+^ per mm^3^: WT 119±21; KO 91±13; *P*=0.051; Fig. 2A). These data indicate that the commissure-forming SATB2^+^ neurons were, in principle, generated in *Tcf4*KO mice. Analysis with the anterograde lipophilic tracer DiI indicated that neurons in *Tcf4*KO mice extended their axons towards the midline yet were unable to cross to the contralateral side (Fig. 1D). Staining for GAP43, a marker for the axonal growth cone, verified this observation (Fig. 2B).

Development and targeting of callosal projections is dependent on the interplay between projection neurons and their environment. Hence, callosal agenesis may not only be the consequence of disrupted developmental programs in projection neurons but can also be the consequence of dysfunctional midline generation and fusion (Richards et al., 2004). Mesman and colleagues (Mesman et al., 2020) recently suggested that loss of callosal projections in their *Tcf4*KO model is caused by the loss of glial wedge glia (also known as midline glia) and failure to form a midline. We analyzed our *Tcf4*KO mice at embryonic day (E) 16.5, to investigate the characteristic detachment of extensions to the pial surface by GFAP-expressing glial wedge glia and the presence of calretinin-expressing guidepost neurons (Paul et al., 2007). Both cell types were detected at the midline in *Tcf4*KO mice and no general defect in the organization of the structure was observed (Fig. 2C,D), suggesting that the midline had been properly formed. Analysis of primary cortical neurons isolated from E16.5 embryos showed that the length of the longest neurite, which will usually be specified into the axon, was significantly reduced in *Tcf4*KO neurons in comparison with WT neurons (longest neurite length in µm: WT 226.02±14.76; KO 176.59±24.45; *P*=0.012; Fig. 2E). This observation suggested that disruption of a TCF4-dependent developmental program in neurons contributed to commissural agenesis in *Tcf4*KO mice.

### *Tcf4* knockout dysregulates genes involved in neuronal differentiation and axon guidance

**Fig. 3.**
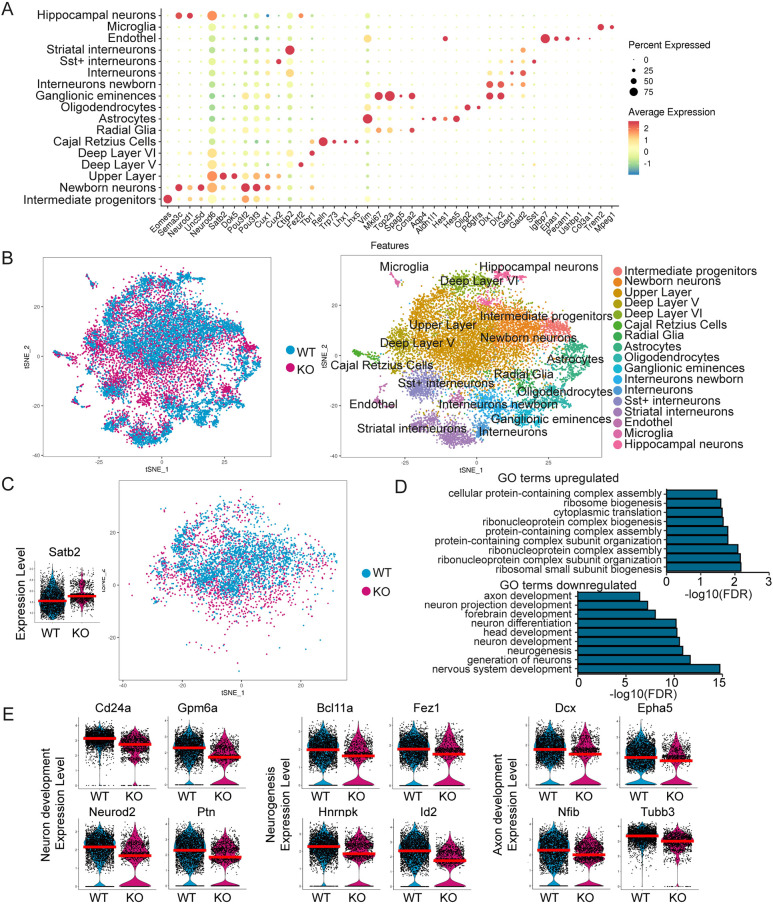
**Single-cell RNA sequencing of E18.5 neocortices from WT and *Tcf4*KO mice.** (A) Dot plot of cell clusters (*y*-axis) and a selection of markers used to assign the cell type (*x*-axis). (B) tSNE plot colored by genotype (left) and cluster identity (right). (C) Right: tSNE plot of *Satb2*-expressing glutamatergic cells used for further analysis. Left: Violin plot of *Satb2* expression in the *Satb2* cluster. The red line depicts the median. (D) Selection of the first 50 GO terms associated with up- and downregulated genes in *Satb2*-expressing glutamatergic cells. GO terms for neurogenesis, neuronal differentiation and axon development were downregulated in the *Tcf4*KO cells. (E) Violin plots of DEGs in the *Satb2* cluster that are associated with neurogenesis and neuron and axon development. The red line depicts the median.

To identify TCF4-dependent networks in the control of forebrain commissure formation, scRNA-Seq was conducted from E18.5 neocortices. After rigorous filtering for viable cells, 8887 cells were analyzed for WT and 5309 for the KO. Both the principal components analysis (PCA) and multiCCA approaches of Seurat were used for cell clustering (Butler et al., 2018; Stuart et al., 2019) and cell types were assigned using known marker expression (Fig. 3A,B, Fig. S2A,B). All expected major cell types were detected and WT and KO cells clustered together regardless of their genotype and of the clustering approach (Fig. 3B, Fig. S2B). Further analyses shown are based on the results of the PCA clustering; downstream analysis with the multiCCA clustering, however, yielded similar results (data not shown).

The focus of our subsequent analyses was *Satb2*-expressing glutamatergic neurons because they represent IPNs that normally cross the CC and AC (Fig. 3C). To define TCF4-dependent networks in commissure-forming neurons, the MAST algorithm was used to determine differentially expressed genes (DEGs) between WT (2890 cells) and KO (1328 cells) *Satb2^+^* neurons (Fig. 3C, Table S1). Upregulated genes (96 genes) were associated with GO terms for ribosomes and gene expression (Fig. 3D). GO terms for downregulated genes (126 genes) included gene enrichments for neuron development (e.g. *Cd24a*, *Gpm6a*, *Neurod2* and *Ptn*), neurogenesis (e.g. *Bcl11a*, *Fez1*, *Hnrnpk* and *Id2*) as well as axon development (e.g. *Dcx*, *Epha5*, *Nfib* and *Tubb3*) (Fig. 3D,E).

Analysis of unique molecular identifiers (UMIs), number of genes and the percentage of mitochondrial genes revealed that KO cells had in general a lower number of expressed genes and UMIs. We therefore limited the data to cells with fewer than 4000 UMIs, yielding 1057 WT and 1246 KO cells (referred to hereafter as limited *Satb2* data set) and re-analyzed DEG and GO term enrichment to control for potential bias introduced by differences in gene and cell number in the *Satb2* cluster. The vast majority of DEGs and GO terms remained the same, indicating the biological relevance of our findings using the full *Satb2* data set (Table S2).

To ascertain that we did not introduce a bias in the analysis by defining the examined cluster only by *Satb2* expression, which appears to be affected by *Tcf4* knockout, we also analyzed the dataset using the upper (UL) and deep layer (DL) cluster that had been assigned based on known marker expression (see Fig. 3A,B). The UL cluster, which mostly consisted of IPNs, was highly comparable to the *Satb2* cluster, with 184 differentially regulated genes shared between the two clusters (*Satb2* DEG 222; UL DEG 206; overlap 184; Fig. S3A-D, Table S3). Moreover, GO terms were similar in UL and *Satb2* clusters (Table S3). For the DL cluster, similar GO terms were identified, but the underlying DEGs diverged considerably from the *Satb2* cluster (*Satb2* DEG 222; DL DEG 165; overlap 111; Fig. S3D-H, Table S4). These results support the validity of our analysis using the *Satb2* cluster to examine *Tcf4*-dependent genetic programs in IPN development.

Collectively, these findings confirm at the molecular level the anatomical observation that *Tcf4* knockout affects the expression of a gene network in *Satb2* neurons/IPNs that is involved in commissure and neuron projection development.

### TCF4 modulates the activity of non-bHLH transcription factors

**Fig. 4.**
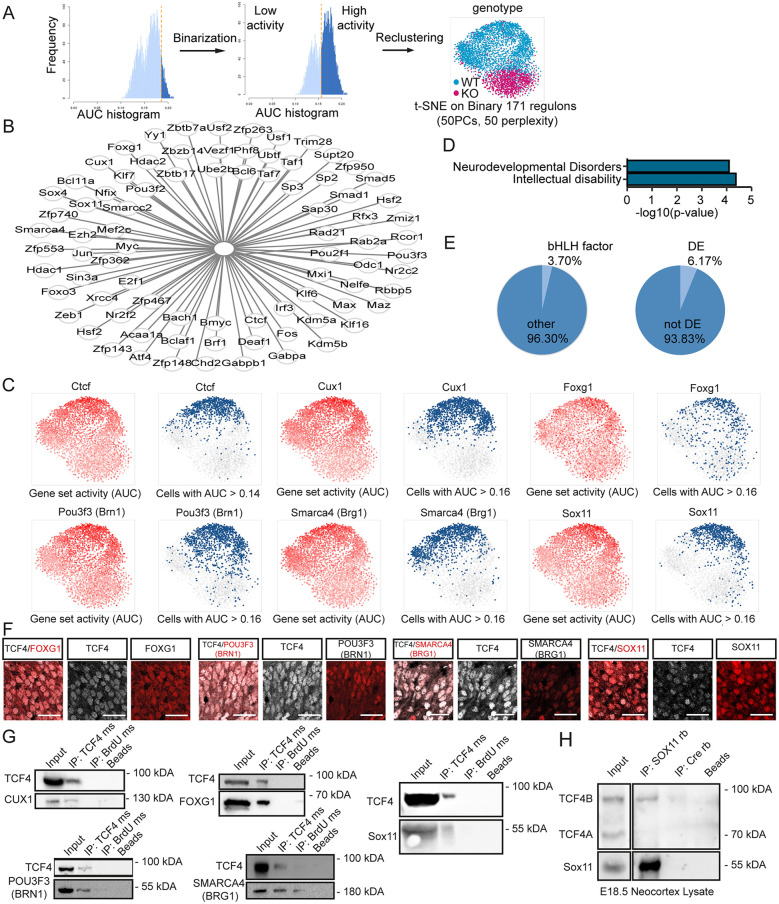
**Gene regulatory network analysis of SATB2-expressing cells.** (A) Scheme of the workflow used to recluster cells after GRN analysis and the resulting tSNE plot of the *Satb2* cluster. Regulons were binarized and reclustered accordingly. WT and KO cells segregated based on GRN activity with only minor overlap. (B) Differentially active regulons of the *Satb2* cluster that may be possible interactors of TCF4. (C) tSNE plots showing the regulon activity of *Ctcf*, *Cux1*, *Foxg1*, *Pou3f3*, *Smarca4* and *Sox11* on a continuous scale (left, red) or binarized (right, blue). The regulons are highly active in the WT cells with only a small number of KO cells showing a high expression. (D) Selection of disease associations enriched in the list of differentially active regulons. (E) Pie charts depicting the percentage of bHLH factors and differentially expressed (DE) regulators in the differentially active regulons. (F) Representative images of TCF4 (white) and FOXG1, POU3F3, SMARCA4 and SOX11 (all in red) immunostaining in the upper third of E18.5 WT cortices. Note the expression in the same nuclei. Scale bars: 50 µm. (G) Co-IP assay using anti-TCF4 antibody conducted with HEK cell extract after overexpression of TCF4 and CUX1, FOXG1, POU3F3, SMARCA4 and SOX11 in HEK cells. The blots presented are cropped. All proteins were co-immunoprecipitated with TCF4, but not with an isotype control for IgG or Agarose A Beads alone except for POU3F3, which was precipitated to a small amount by the isotope control IgG. The interactions were confirmed in three independent biological replicates. ms, antibody raised in mouse. (H) Co-IP assay conducted with E18.5 cortex lysates using anti-SOX11 antibody. Upper panel: detection with anti-TCF4 antibody. Lower panel: detection with anti-SOX11 antibody. The blots presented are cropped. TCF4B was co-immunoprecipitated with SOX11, but not with an isotype control for IgG and Agarose A Beads alone. The interaction was confirmed in three independent biological replicates. rb, antibody raised in rabbit.

We next aimed to investigate the influence of TCF4 on gene regulatory networks (GRNs) using the R package SCENIC. Identified regulons were binarized to generate a high/low activity state (Fig. 4A). Re-clustering displayed a partitioning of cells between the two genotypes with only marginal overlap (Fig. 4A). Regulons were sorted into three categories: (1) regulon primarily active in the WT; (2) no differential activity between the genotypes; and (3) regulon predominantly active in the KO. Differentially active regulons mostly fell into the first category [e.g. *Ctcf*, *Foxg1*, *Smarca4* (also known as *Brg1*), *Cux1*, *Pou3f3* (also known as *Brn1*) and *Sox11*] (Fig. 4B,C, Table S5). As with the DEG analysis, GRN analysis was also performed with the limited *Satb2*, UL and DL datasets. In the limited *Satb2* (Table S5) and the UL dataset we found, similar to our analyses of the full *Satb2* dataset, IPN-specific TF-dependent regulons such as *Pou3f3* and *Cux1* being differentially active (Fig. S4A-F, Table S5). In contrast, differentially active regulons in the DL cluster represented TFs known to be involved in deep layer development, e.g. *Sox5* or *Foxp2* (Fig. S4G-L, Table S5). Further analysis was therefore done with the original *Satb2* dataset.

Examination of the differentially active regulons revealed that most regulon heads were TFs. Moreover, we found an enrichment for TFs associated with intellectual disability (e.g. *CTCF*) and neurodevelopmental disorders (e.g. *FOXG1*) (Fig. 4D) (Gregor et al., 2013; Kortum et al., 2011). Surprisingly, these TFs generally did not belong to the bHLH TF family, which constitutes the canonical interaction partners of TCF4 (bHLH factors: 3.70%; other: 96.30%) (Fig. 4E). In addition, the vast majority of regulators were not differentially expressed (DE) in the KO (DE: 6.17%; not DE: 93.83%) (Fig. 4E), suggesting that they were not downstream targets of TCF4. A recent *in vitro* study suggested that TCF4 may interact with TFs outside the bHLH family (Moen et al., 2017). In accordance, we hypothesized that TCF4 interacts with the identified non-bHLH TFs and modulates their activity. We focused on potential interactions regulating genes involved in neurogenesis or neuron differentiation. To ensure a focus on robustly expressed regulons, the selected regulators had to be expressed in at least a quarter of the *Satb2^+^* cells (Table S5). Five regulon heads (*Foxg1*, *Smarca4*, *Cux1*, *Pou3f3* and *Sox11*) were selected for validation (Fig. 4C). In the context of postmitotic neurons of the cortical plate, CUX1 and POU3F3 are specifically expressed in layer II/III neurons, whereas FOXG1, SMARCA4 and SOX11 are broadly expressed during neuronal differentiation (Bergsland et al., 2006; Miyoshi and Fishell, 2012; Molyneaux et al., 2007; Deng et al., 2015). Staining of neocortical tissue at E18.5 showed that TCF4 was co-expressed with SOX11, FOXG1, SMARCA4 and POU3F3 (Fig. 4F). *In vitro* co-immunoprecipitation assays (co-IPs) confirmed that the long TCF4 isoform has the potential to interact biochemically with all these TFs (Fig. 4G) as did proximity ligation assays in 6 day differentiated neural stem cells derived from E14.5 neocortices (Fig. S5). Collectively, these results indicate that TCF4 has the ability to interact biochemically with a wide variety of TFs and chromatin remodelers involved in neurogenesis and neuronal differentiation, and suggests that these interactions are relevant for the precise execution of the developmental transcriptional program in *Satb2^+^* cortical neurons.

**Fig. 5.**
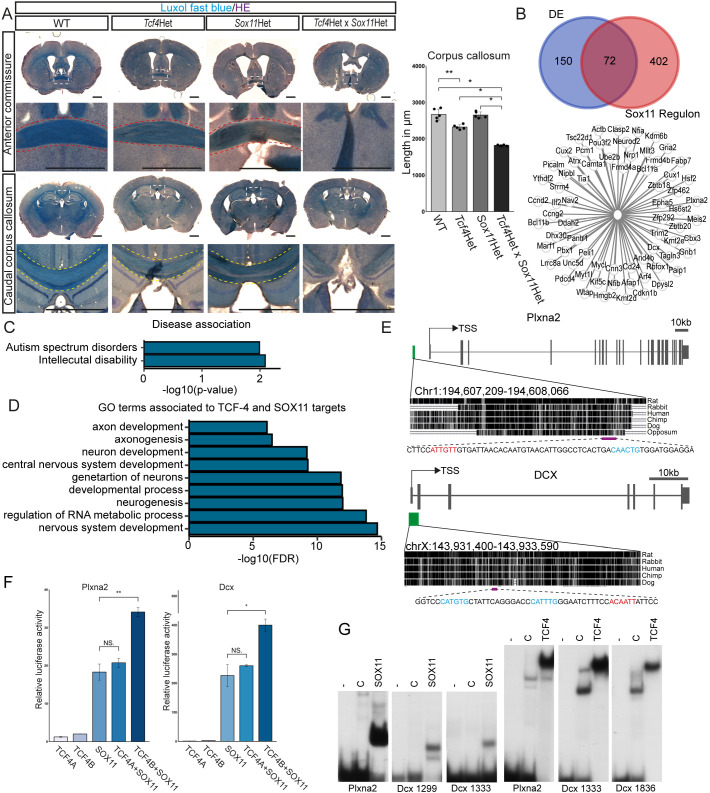
**TCF4 and SOX11 act synergistically in corpus callosum formation.** (A) Representative overview images of Luxol Fast Blue staining at the position of the AC and the caudal body of the CC. Images below the overview images are magnifications of the boxed areas. Yellow dashed lines indicate the CC crossing the midline. Red dashed lines indicate the AC. In *Tcf4* and *Sox11* double haploinsufficient mice, agenesis of the AC and agenesis of the splenium and caudal part of the body of the CC can be observed. Quantification of slices showing a CC is presented on the right (*n*=5, *P*-values were determined with Mann–Whitney-U tests; mean±s.d., WT 2664±137.64 µm; *Tcf4*Het 2320±56.57; *Sox11*Het 2648±77.56; *Tcf4*Het×*Sox11*Het 1816±19.60; WT versus *Tcf4*Het: *P*=0.008; WT versus *Tcf4*Het×*Sox11*Het: *P*=0.011; *Tcf4*Het versus *Tcf4*Het×*Sox11*Het: *P*=0.011; *Sox11*Het versus *Tcf4*Het×*Sox11*Het: *P*=0.011). Scale bars: 1000 µm. For more overview and magnification images, see Fig. S7. (B) Venn diagram of the overlap of DEGs and the targets of the *Sox11* regulon in the *Satb2* cluster. Possible common targets are depicted below. (C) Selection of disease associations enriched in the list of common targets of TCF4 and SOX11 in the *Satb2* cluster. (D) Selection of GO terms associated with the common targets of TCF4 and SOX11 in the *Satb2* cluster. GO terms for neurogenesis, neuronal differentiation and axonogenesis were enriched. (E) Schematic of *Plxna2* and *Dcx* genes and the location of the ECRs with TCF4- and SOX11-binding capacity (green box) relative to the transcriptional start site (TSS). The magenta bar indicates the regions with possible binding sites for TCF4 (blue) and SOX11 (red). For DNA sequences, see Fig. S9. (F) Relative luciferase reporter gene activity under the control of the regulatory regions of *Plxna2* and *Dcx* in transiently transfected HEK cells co-expressing TCF4A, TCF4B, SOX11 and a combination of either TCF4A or TCF4B with SOX11 (*n*=3, *P*-values were determined with two-tailed Student's *t*-tests; presented as mean±s.d., transfection with empty CAG-GFP vector was set to 1 for each regulatory region; *Dcx*: SOX11 versus TCF4A+SOX11: *P*=0.387; SOX11 versus TCF4B+SOX11: *P*=0.006; *Plxna2*: SOX11 versus TCF4A+SOX11: *P*=0.462; SOX11 versus TCF4B+SOX11: *P*=0.025). (G) For electrophoretic mobility shift assay, oligonucleotides containing potential SOX11-binding sites and E-Boxes from the *Plxna2* and *Dcx* ECRs (for sequences, see Fig. 5E or Fig. S9) were incubated without cell extract (−), or in the presence of extracts from HEK293 cells transfected with empty expression vector (‘C’) or expression vector for the SOX11 high mobility group domain (amino acids 38-126 of SOX11) or the bHLH domain of TCF4B (amino acids 491-617 of TCF4B).

We sought to genetically validate the functional relevance of the interaction between TCF4 and SOX11 in commissural development *in vivo*. Co-IPs from E18.5 neocortex lysates confirmed the interaction of TCF4B with SOX11 *in vivo* (Fig. 4H). Notably, we did not observe binding of SOX11 to TCF4A in the *in vivo* co-IPs, although TCF4A was detectable in the input (Fig. 4H). Next, *Tcf4* and *Sox11* haploinsufficient mice were crossed to generate WT, *Tcf4*, *Sox11* and double *Tcf4*×*Sox11* haploinsufficient littermates. Commissural systems in P56 brains were visualized by Luxol Fast Blue staining. WT and *Sox11* haploinsufficient mice showed no commissural phenotype (Fig. 5A). As previously reported (Jung et al., 2018), *Tcf4* haploinsufficient animals had a mildly shorter CC. This phenotype was greatly aggravated by the additional haploinsufficiency of *Sox11* as double haploinsufficient mice showed the most severe truncation of the CC with only the rostral part of the CC remaining (agenesis of the splenium and caudal part of the body) (WT 2664±137.64 µm; *Tcf4*Het 2320±56.57 µm; *Sox11*Het 2648±77.56 µm; *Tcf4*Het×*Sox11*Het 1816±19.60 µm; WT versus *Tcf4*Het: *P*=0.008; WT versus *Tcf4*Het×*Sox11*Het: *P*=0.011; *Tcf4*Het versus *Tcf4*Het×*Sox11*Het: *P*=0.011; *Sox11*Het versus *Tcf4*Het×*Sox11*Het: *P*=0.011) (Fig. 5A, Fig. S6). Moreover, only a rudimentary AC (1/5 animals) or a complete agenesis of the AC (4/5 animals) was observed in the double haploinsufficient mice (Fig. 5A, Fig. S6). We also performed analyses at P7 when all commissural axons have crossed the midline and started innervating the contralateral hemispheres, to determine whether the commissural phenotype was caused by maldevelopment or by degeneration of IPNs or their projections. Double haploinsufficient mice showed a severely truncated CC and loss of the AC at P7 (Fig. S7), indicating that the combined decrease in *Sox11* and *Tcf4* gene dosage impaired commissural system development.

We next investigated which genes may be common target genes of both TFs and thus may be involved in commissure development. We compared the predicted regulon targets of SOX11 from the GRN analysis and the list of DEGs in the *Tcf4KO*. Comparison of the datasets yielded a list of 72 genes (Fig. 5B, Table S6), which was enriched for genes linked to autism spectrum disorders (e.g. *POU3F2*) and intellectual disability (e.g. *DCX*) (Fig. 5C) (Pilz et al., 1998; Martin et al., 2007). Furthermore, GO term analysis revealed an enrichment for genes associated to axonogenesis (Fig. 5D, Table S5). *Plxna2*, a gene involved in semaphorin/plexin signaling for axon guidance (Rohm et al., 2000; Mitsogiannis et al., 2017), and *Dcx*, a gene that in addition to its function in neuronal migration has been connected to neuronal morphogenesis and axon guidance (Deuel et al., 2006; Pilz et al., 1998; Yap et al., 2016; Bott et al., 2020), were selected for further investigation. Evolutionarily conserved regions (ECRs) upstream of or at the promotor containing conserved binding sites for TCF4 and SOX11 (Fig. S8) were cloned into luciferase reporter plasmids and transfected into HEK293T cells together with expression plasmids for TCF4 and SOX11 (Fig. 5E). SOX11 alone induced robust *Dcx* and *Plxna2* reporter activity. TCF4A or TCF4B alone only marginally induced *Plxna2* and *Dcx* activity but TCF4B strongly potentiated SOX11-induced reporter activities (Fig. 5F), supporting the general notion that Sox factors pair with other TFs to induce specific gene expression strongly (Kamachi et al., 2000; Reiprich and Wegner, 2015). In contrast, no change in activity over SOX11 alone was seen when TCF4A was co-expressed with SOX11 (Fig. 5F). To ascertain whether TCF4 and SOX11 have the potential to bind to the determined regulatory regions of *Plxna2* and *Dcx* (Fig. 5E), we performed electrophoretic mobility shift assays. We were able to show the binding of both the TCF4 and the SOX11 DNA-binding domain to the regulatory regions of *Plxna2* and *Dcx* (Fig. 5G). Collectively, these data support the hypothesis that TCF4 and SOX11 can cooperate to regulate transcription of common target genes.

In summary, these findings indicate that TCF4 and SOX11 cooperate in the regulation of AC and CC formation potentially by activating expression of genes related to axonogenesis and axon guidance. These results also provide proof of principle that the scRNA-Seq-based strategy presented here is suitable for the identification of functionally relevant TCF4 interactors in a defined neuronal population.

## DISCUSSION

Here, we provide scRNA-Seq and biochemical evidence that the transcription factor TCF4, which has been linked with schizophrenia, autism and intellectual disability, engages in non-canonical interactions with non-bHLH TFs to regulate the development of interhemispheric connectivity. Interestingly, a substantial fraction of TCF4-interacting transcription factors has also been associated with intellectual disability and autism, raising the possibility that the TCF4-dependent regulatory network in commissure formation may be relevant for the pathogenesis of neurodevelopmental and neuropsychiatric disorders.

Here, we analyzed *Tcf4* knockout mice generated by the homozygous insertion of a *lacZ*/neomycin cassette before exon 4 of *Tcf4*. This mutation disrupts the expression of longest TCF4 isoform, TCF4B, but leaves shorter isoforms, such as TCF4A, intact. Notably, the phenotype of our *Tcf4*KO mice is comparable with the phenotype of TCF4 homozygous KO mice harboring a loss-of-function mutation affecting the DNA-binding bHLH domain (Mesman et al., 2020; Li et al., 2019), strongly indicating that TCF4B represents the isoform that is required for cortical layering and commissural development. The disparity regarding the midline formation between our study and the study by Mesman and colleagues may in principle be caused by the different *Tcf4* knockout strategies and the remaining expression of shorter isoforms with DNA-binding potential. It is, however, important to keep in mind that the midline was analyzed at different developmental time points; whereas we performed our analyses at E16.5 – the time-point at which the interhemispheric fissure is fusing, acute midline remodeling is occurring and pioneer axons are crossing into the contralateral hemisphere, Mesman and colleagues analyzed the midline at P0, when interhemispheric fissure remodeling is mostly completed (Gobius et al., 2016).

The present knockout model allows for the expression of shorter TCF4 isoforms with an intact bHLH DNA-binding domain, such as TCF4A. Reporter assays of genes that were predicted to be regulated by cooperation of TCF4 with SOX11 indicated (1) that cooperativity with SOX11 was specific to TCF4B, and (2) that TCF4A did not impair SOX11-dependent transcriptional activity. Furthermore, *in utero* overexpression of TCF4A at E13.5 had no adverse impact on cortical layer generation (Fig. S9A). TCF4A overexpressing neurons were also capable of crossing the midline (Fig. S9B). These data indicate that the cortical layering phenotype and loss of intercortical projections is caused by the loss of the longest TCF4 isoform and not by the residual expression of TCF4A. Together with the similarity in phenotypes between the present knockout mice and the TCF4 KO mice generated by disruption of the bHLH domain, these findings indicate that the remaining expression of shorter isoforms such as TCF4A neither ameliorates nor aggravates the loss-of-TCF4B phenotype.

The generation of SATB2^+^ neurons was not abolished by the loss of TCF4, indicating that the loss of the commissural system in *Tcf4*KO mice is not the result of a failure to generate interhemispheric projection neurons. As discussed, we could not corroborate the notion of Mesman et al. (2020) that *Tcf4*KO impairs midline formation, but it is important to note that our histological analyses cannot exclude subtle defects in midline development and axon guidance cues. Our finding of defective neurite outgrowth in isolated *Tcf4*KO cortical neurons, however, indicate that *Tcf4*KO directly perturbs neurodevelopmental programs in cortical neurons.

To understand further the TCF4-dependent genetic programs in intercortical projection neurons, we performed RNA-sequencing analysis. Previous bulk RNA-sequencing analyses had shown that TCF4 regulates a diverse set of genes with functions in cell proliferation, neuronal differentiation and neurotransmitter release (Li et al., 2019; Mesman et al., 2020). Given the broad expression of TCF4 (Jung et al., 2018) and the considerable cellular diversity in the developing neocortex, available bulk RNA-sequencing data were not ideally suited for identification of cell type and or stage-specific TCF4-dependent mechanisms. We therefore used comparative single-cell RNA-sequencing analysis to zoom in onto the TCF4-dependent transcriptome in *Satb2*-expressing neurons. This analysis not only allowed us to uncover potential TCF4 targets, but, more importantly, also enabled the prediction of TCF4-interacting transcription factors in a cell type-specific manner. Surprisingly, we found evidence that TCF4 interacts with a multiplicity of TFs and chromatin remodelers other than the classical interaction partners of the bHLH family.

In our new approach, prediction of functional transcription factor interaction was based on differential regulon activity. As a caveat, divergence in the activity of regulons may be a result of unbalanced cellular coverage between genotypes during sequencing. The SCENIC algorithm, however, is considered to be relatively resistant to dropouts (Aibar et al., 2017). Furthermore, we present *in vitro* biochemical evidence for several of the predicted interactions and provide *in vivo* biochemical and genetic evidence for the cooperativity of TCF4 with the non-bHLH transcription factor SOX11 in the generation of the commissural system. We identified two potential common target genes of TCF4 and SOX11, *Dcx* and *Plxna2*, which have been shown to play important roles in axonal development and axon guidance (Rohm et al., 2000; Mitsogiannis et al., 2017; Deuel et al., 2006; Pilz et al., 1998; Yap et al., 2016; Bott et al., 2020) and are likely candidates to contribute to the observed dysgenesis of the forebrain commissures in *Tcf4*KO mice. These two genes are, however, only two examples out of a larger network of factors involved in commissural formation that are dysregulated as a result of changes in GRN activities caused by the loss of TCF4B.

Future studies should determine which domains mediate the interaction of TCF4 with non-bHLH transcription factors. Previous analysis had revealed the existence of multiple TCF4 isoforms (Sepp et al., 2011), with TCF4A (short isoform) and TCF4B (longest isoform) being the two main isoforms. Both the TCF4A and TCF4B isoforms were detected in protein isolates from the embryonic mouse cortex; however, only TCF4B co-immunoprecipitated with the non-bHLH transcription factor SOX11. We were also able to show that TCF4B does not impede TCF4A binding to SOX11 as TCF4A was not co-immunoprecipitated with SOX11 when using protein isolates from the neocortex of KO mice (Fig. S10C). Moreover, the present *Tcf4*KO mouse model specifically lacks the TCF4B isoform, indicating that the dysregulation of non-bHLH TF activity in these mice is dependent on TCF4B-specific domains. Collectively, our data strongly suggest that the interaction of TCF4 with non-bHLH transcription factors may be conferred by the TCF4B-specific N-terminal domain.

Current evidence suggests that CC dysgenesis significantly contributes to cognitive impairment and associative dysfunction in intellectual disability, autism and schizophrenia (Bedeschi et al., 2006; Paul et al., 2007; Rao et al., 2011; Siffredi et al., 2013; Badaruddin et al., 2007). Intriguingly, several of the predicted TCF4 interactors have been linked to neurodevelopmental disorders featuring corpus callosum abnormalities (Pringsheim et al., 2019; Filatova et al., 2019; Tzeng et al., 2014; Snijders Blok et al., 2019; Hempel et al., 2016), suggesting a pathophysiological relevance of the proposed TCF4 TF network.

The present data may therefore provide a new entry point towards understanding central dysregulated networks in the pathogenesis of neurodevelopmental disorders. Additionally, the present study indicates that scRNA-Seq data can be harnessed to predict interaction partners of proteins. This powerful approach may prove valuable to infer cell type-specific TF networks from complex tissues, thereby enabling the discovery of regulatory networks in development, physiology and disease.

## MATERIALS AND METHODS

### Experimental models

All experiments were carried out in accordance with the European Communities Council Directive (86/609/EEC) and were approved by the government of Middle-Franconia. Tcf4ex4WT/lacZ mice (MGI ID: 4432303) (Jung et al., 2018) and Sox11^LacZ/WT^ mice (Sock et al., 2004) were described previously.

Experiments were performed on male and female littermates at E16.5, E18.5, P0, P7 and P56. For embryonic studies, mice were bred in the afternoon and vaginal post-coitum protein plug check was performed the next morning (defined as E0.5). Numbers of animals used in each experiment are indicated in the figure legends.

Genotyping of the mice was performed using the following primers: Tcf4ex4WT/lacZ: fwd Mut TCGTGGTATCGTTATGCGCC, fwd WT CCGATGACAGTGATGATGGT, rev AAGTTAAGCTGAAGTAAATACCCACA, lacZ fwd ATCACGACGCGCTGTATC, lacZ rev ACATCGGGCAAATAATATCG; Sox11^LacZ/WT^: fwd GCCCGCGCAGGAGACCGAGC, rev CTTGTAGTCGGGGTAGTCAGCC, lacZ CGCTCAGGTCAAATTCAGAC.

### Tissue preparation and dissection

Timed pregnant mice were killed by cervical dislocation. For the E16.5, E18.5 and P0 time points, brains were dissected and fixed overnight in 4% paraformaldehyde (PFA). Tails were used for genotyping. After fixation, tissue was transferred to 30% sucrose in 0.1 M phosphate buffer overnight for dehydration. Embryonic tissues were embedded in freezing media (Leica Biosystems) and stored at −80°C. P7 mice and adult mice (P56) were killed using CO_2_ and transcardially perfused with PBS for 2 min (20 ml/min) followed by 4% PFA in PBS, pH 7.4, for 5 min. The brains were post-fixed overnight in 4% PFA at 4°C followed by dehydration at 4°C in 30% sucrose in 0.1 M phosphate buffer.

### Histology

Embryonic tissue was cut serially in 10 μm thin sections with a cryotome (Leica Microsystems). Sections were transferred onto laminated object slides, dried for 2 h at room temperature (RT) and stored at −80°C. Slides were washed once for 5 min with 1× PBS. For antigen retrieval, sections were treated with 10 mM citrate buffer (pH 6) for 11 min at 720 W in the microwave. Half of the citrate buffer was replaced by water and the sections were immersed at room temperature for another 30 min. Slides were washed once in 1× PBS and then incubated in 4% PFA for 10 min followed by two more washing steps in 1× PBS. Tissue was permeabilized for 10 min in 0.3% Triton X-100/PBS and blocked with blocking solution [10% donkey serum, 3% bovine serum albumin (BSA) and 0.1% Tween 20 in PBS] for 1 h in a wet chamber at RT. Sections were incubated with primary antibodies [rabbit anti-BRN1 (kind gift of Elisabeth Sock; Schreiber et al., 1997; 1:500); rabbit anti-BRG1 (Santa Cruz Biotechnology, sc10768; 1:100); rabbit anti-calretinin (Swant, 7699/4; 1:500); rabbit anti-CTIP2 (Abcam, 18465; 1:500); rabbit anti-FOXG1 (Abcam, ab18259; 1:500); rabbit anti-GAP43 (Abcam, ab5220; 1:500); chicken anti-GFAP (Abcam, ab4674; 1:500); mouse monoclonal SATB2 (Santa Cruz Biotechnology, sc-81376; 1:500); rat anti-SOX11 (kind gift from Johannes Glöckner, Deutsches Zentrum für Neurodegenerative Erkrankungen, Tübingen, Germany; 1:500); rabbit anti-TBR1 (Abcam, ab31940; 1:500); mouse monoclonal TCF4 (Santa Cruz Biotechnology, sc393407; 1:100)] diluted in blocking solution at 4°C overnight. Slides were washed three times for 5 min with 0.1% Tween/PBS, incubated with secondary antibodies diluted in blocking solution for 2 h at RT, and washed three times with 1× PBS. Nuclei were stained with DAPI (500 pg/ml in 1× PBS) for 10 min. After additional washing with 1× PBS for 5 min, slides were mounted with 60 μl Mowiol (Sigma-Aldrich) and stored at 4°C.

### Cell counting

Cell counting was performed blind to avoid bias. Numbers were randomly assigned to slides before imaging. Genotypes were only revealed for statistical analysis. All images were taken with the pial surface at the upper edge of the picture and the ventricular surface at the lower edge. Cells were counted using ImageJ software and reported as the density of marker-positive cells per mm^3^ of cortical area counted. For each animal, one rostral and caudal section of the neocortex was counted.

### Lipophilic tracer analysis

For the lipophilic tracer experiment, P0 brains were dissected and washed once in 1× PBS. 1 µl of DiI dilution [DiIC18(3), Invitrogen] was pipetted onto one hemisphere and the brain fixed in 4% PFA. After 6 weeks, the tissue was transferred to 30% sucrose in 0.1 M phosphate buffer overnight. Brains were sectioned as described in the Histology section. For histology, the antigen retrieval and antibody incubation steps were omitted. Nuclear counterstaining with DAPI was conducted as described in the Histology section.

### Primary cortical neuron culture and neurite measurement

To generate primary cortical neuron cultures, timed pregnant mice were killed by cervical dislocation at E16.5 and the neocortices of single embryos were dissected under a binocular and immediately transferred to ice-cold HBSS (Gibco). Tails were used for genotyping. After removal of HBSS, the tissue was incubated in 2 ml dissociation medium [HBSS, 0.01% Papain (Sigma-Aldrich), 0.1% Dispase II (Roche), 0.01% DNAse I (Roche) and 12.4 mM MgSO_4_] for 10 min at 37°C, after which the solution was then gently triturated ten times with a glass pipette. This step was repeated two more times. Cells were strained with a 70 µm strainer, which was washed with 1 ml of NBA-Mix [Neurobasal Medium (Gibco), 0.2 mM GlutaMax (Gibco), 1× Neurobrew-21 (MACS Miltenyi Biotec), 0.1 M sodium pyruvate (Gibco) and 0.1 M Antibiotic-Antimycotic (Gibco)]. The solution was centrifuged for 5 min at 120 ***g*** and then washed with 1 ml NBA-Mix and centrifuged again. This step was repeated once more then 200,000 cells were seeded on PDL/laminin-coated coverslips in 24-well plates. Cells were fixed after 3 days *in vitro* and stained with rabbit anti-TUBB3 (Abcam, 18207; 1:500), mouse monoclonal SATB2 (Santa Cruz Biotechnology, sc-81376; 1:500), rabbit anti-CTIP2 (Abcam, 18465; 1:500) and DAPI. Single neurons, which did not contact other neurons in the vicinity and only expressed SATB2, but not CTIP2, were imaged for analysis to ensure that only intercortical projection neurons were compared. Twenty cells per animal were analyzed. Longest neurite lengths were measured from the soma to the tip of the longest neurite using the Simple neurite tracer tool of Fiji (Longair et al., 2011).

### Luxol Fast Blue staining

Brains were serially cut into 40 µm sections. Free-floating sections were washed twice with 1× PBS, mounted on coated adhesive glass slides and dried for at least 2 h at RT. The glass slides were incubated in Luxol Fast Blue solution (Polyscience) at 57°C overnight and washed once in 95% ethanol and once in distilled water. The staining was differentiated in lithium carbonate solution for 3 min followed by incubation in 70% ethanol until white and gray matter were distinguishable. The nuclei were stained with Mayer's Hemalun solution for 30 s and rinsed with tap water. Slides were mounted with 60 μl Mowiol and stored at 4°C. To quantify the length of the corpus callosum, the slices were counted from the first slice to the last slice showing a corpus callosum.

### Imaging

For overview images, cell counting and neurite length measurement, fluorescence signal was detected with an AF6000 Modular Systems Leica fluorescent microscope and documented with a SPOT-CCD camera and Leica software LAS AF (Version 2.6.0.7266; Leica Microsystems). For analysis of the Luxol Fast Blue staining, images were obtained with a Zeiss MN Imager and ×2.5 objective lens. For co-expression analysis, fluorescence signal was detected using a Zeiss LSM 780 confocal microscope with four lasers (405, 488, 550 and 633 nm) and ×40 objective lens. Images were processed using ImageJ.

### Co-IP

For *in vitro* co-IP HEK 293T cells (ATCC; CRL-3216) were seeded in 10 cm dishes in DMEM supplemented with 10% of fetal bovine serum and 5 ml penicillin/streptomycin. At a confluency of 70-90%, cells were transfected using JETPEI (Polyplus transfection, 101-10N) with equal amounts of the expression vectors (7.5 µg/10 cm dish) of CAG-TCF4-IRES-GFP and the predicted interaction partners [pCMV5 rBrn1 (Schreiber et al., 1997); pBJ5-hBRG1 (a gift from Jerry Crabtree; Addgene plasmid #17873) (Khavari et al., 1993); pXJ42-p200 CUX1 (a gift from Alain Nepveu; Addgene plasmid #100813; Wilson et al., 2009); CAG-*Foxg1*-IRES-RFP; CAG-*Sox11*-IRES-GFP (Balta et al., 2018)] according to the manufacturer's instruction. After 48 h, three 10 cm dishes were harvested in 1 ml Buffer A [10 mM HEPES, pH 7.9, 10 mM KCl, 0.1 mM EDTA, pH 8.0, 0.1 mM EGTA, pH 8.0, protease inhibitor EDTA-free cocktail (Roche PVT) and phosphatase inhibitors cocktail (Sigma-Aldrich)]. After addition of 100 µl of 10% NP-40 and 84 µl of 5 M NaCl, the solution was vortexed followed by 15 min of incubation on a rotating wheel at 4°C. The homogenates were centrifuged at 14,000 ***g*** for 3 min. The supernatant was used directly for the co-IP by mixing 300 µl with 1.2 ml of TEN-Buffer [10 mM Tris, pH 7.4, 0.05 mM EDTA, 50 mM NaCl, 0.25% 10% NP40, protease inhibitor EDTA-free cocktail and phosphatase inhibitors cocktail] and 2 µl of mouse monoclonal TCF4-antibody (Santa Cruz Biotechnology, sc393407), 2 µl of mouse monoclonal BrdU-antibody (BD Bioscience, B44) (control for nonspecific binding to mouse antibodies), or nothing to control for nonspecific binding to the beads. An appropriate amount of the supernatant was kept as input. Probes were incubated on a rotating wheel at 4°C overnight then 30 µl of Protein A Agarose Beads, Fast Flow (Millipore-Merck) in TEN-Buffer (1:1) were added and the samples were rotated for another 3 h at 4°C. Samples were centrifuged for 5 min at 1200 ***g*** and the supernatant was discarded. Beads were washed three times with 500 µl of TEN-Buffer and frozen at −80°C. For western blot analysis, 30 µl of 3× Laemmli buffer was added to samples, which were incubated at 95°C for 5 min then 25 µl of the samples were loaded on 10% SDS gels.

For *in vivo* co-IP, neocortices of E18.5 WT embryos were dissected. Two cortices were homogenized in 1 ml of Buffer A. Samples were treated as described above. Antibodies used were 1 µl rabbit anti-SOX11-antibody (Abcam, ab134107) and 1 µl rabbit anti-Cre-antibody (Abcam, ab110465). For western blot analysis, 50 µl of 3× Laemmli buffer was added to the beads, which were incubated at 95°C for 5 min then 20 µl of the samples were loaded on 10% SDS gels or 4-12% Bis-Tris gels.

### Western blot

Protein extracts from E18.5 WT or KO cortices were obtained by homogenizing the tissue in RIPA buffer [50 mM Tris-HCl, pH 8.0, 150 mM NaCl, 1% Nonidet P-40, 0.5% sodium deoxycholate, 0.1% SDS, 2 mM EDTA, protease inhibitor EDTA-free cocktail and phosphatase inhibitors cocktail] followed by incubation for 30 min on ice. The post-nuclear supernatant of the lysate was obtained by centrifugation at 2000 ***g*** for 10 min at 4°C. Protein content was measured using the Pierce BCA protein assay (Thermo Scientific). Thirty micrograms of protein were loaded on a 10% SDS-PAGE gel. Gels underwent wet transfer onto a nitrocellulose membrane. Membranes were blocked in PBS with 0.1% Tween 20 (PBS-T). Incubation with primary antibodies [rabbit anti-BRN1 (a kind gift from Elisabeth Sock; Schreiber et al., 1997; 1:500); rabbit anti-BRG1 (Santa Cruz Biotechnology, sc10768; 1:500); rabbit anti-FOXG1 (Abcam, ab18259; 1:500); rat anti-SOX11 (a kind gift from Johannes Glöckner, Deutsches Zentrum für Neurodegenerative Erkrankungen, Tübingen, Germany; 1:500); mouse monoclonal TCF4 (Santa Cruz Biotechnology, sc393407; 1:500); mouse monoclonal CUX1 (Abcam, ab242194, 1:500); anti-mouse HRP (Jackson Immuno Research, 115-035-003; 1:10000); anti-rat HRP (Jackson Immuno Research, 112-035-003; 1:10000); and Protein A-HRP (ThermoFisher Scientific, 1:12000)] diluted in 5% BSA in PBS-T was performed overnight at 4°C and was followed by three washes with PBS-T. Secondary antibodies were diluted in PBST and incubated with the membranes for 1 h at RT followed by washing with PBS-T. Membranes were treated with Clarity Western Enhanced Chemiluminescence Substrate (Bio-Rad) and visualized with Fusion-SL (PeqLab). Images were processed via Fusion (PeqLab).

### Luciferase assay

The ECR from the *Plxna2* gene had the following positions in Mm10: chr1:194607209-194608066 (Fig. S9). The ECR was obtained by PCR from WT mouse DNA and inserted into the pTATA luciferase reporter plasmid between XhoI and SacI sites in front of a β-globin minimal promoter (the plasmid was based on Kuhlbrodt et al., 1998). The hDCX-Promotor plasmid has been described before by Karl et al. (2005). In short, the 3509 bp regulatory region (Fig. S9) of the *DCX* promotor was subcloned into a vector (pGl3-Basic) containing the gene for firefly luciferase (Promega).

HEK cells were seeded at a density of 80,000 cells per well in a 24-well plate and transfected the next day. In each well, 300 ng of CAG-GFP-based expression vectors in total [CAG-GFP; CAG-*Sox11*-IRES-GFP (Balta et al., 2018); CAG-TCF4A-IRES-GFP, CAG-TCF4B-IRES-GFP, 100 ng of luciferase reporter (hDCX-pGL3 (Karl et al., 2005) or pTataLuc-Plxna2] and 10 ng of *Renilla* expression plasmid were transfected using JETPEI (Polyplus transfection, 101-10N) according to the manufacturer’s instructions. Three independent biological replicates were performed, with three technical replicates for each condition per biological replicate. After 48 h, the luciferase assay was performed according to the manufacturer’s instructions using the Dual-Luciferase Reporter Assay System Kit (Promega).

### Electrophoretic mobility shift assays

Protein extracts were obtained by transfecting HEK293 cells with JETPEI (Polyplus transfection, 101-10N) using 5 µg expression plasmid per 10 cm plate, and harvested 48 h post-transfection with Buffer A as described in the co-IP section. pCMV5, pCMV5-Sox11-HMG (coding for amino acids 38-126 of SOX11) and pCMV5-TCF4-bHLH-T7 (coding for amino acids 491-617 of the TCF4B isoform) were used for transfection.

Electrophoretic mobility shift assays were performed in the presence of 1 μg poly-dGdC (SOX11) or 0.3 μg sheared salmon sperm DNA (100-400 bp) (TCF4) as a nonspecific competitor using ^32^P-labeled 25- to 52 bp-long double-stranded oligonucleotides and HEK293 whole-cell extracts (Kuhlbrodt et al., 1998). Oligonucleotides contained putative *Sox11*-binding sites or E-boxes identified in the ECRs (see Fig. 5E).

### Proximity ligation assay

WT neurospheres derived from the subventricular zone of E14.5 neocortices were used for all experiments. To generate neurosphere cultures, time-pregnant mice were killed by cervical dislocation at E14.5 and neocortices of single embryos were dissected under a binocular and immediately transferred to ice-cold PBS. After removal of PBS, the tissue was incubated in 5 ml dissociation medium (1× HBSS, 5.4 mg/ml glucose, 15 mM HEPES, trypsin, hyaloronidase) for 15 min at 37°C. The solution was then gently triturated ten times with a glass pipette and incubated for another 15 min at 37°C. 5 ml of ice-cold solution 3 (4% BSA, 20 mM HEPES in EBSS) was added, followed by passage through a 70 μm strainer and centrifugation at 300 ***g*** for 5 min. The supernatant was discarded and cells were resuspended in 10 ml of ice-cold solution 2 (0.5× HBSS, 90 mM sucrose). The solution was centrifuged again for 10 min at 400 ***g***, the supernatant was removed and cells were mixed with 2 ml of ice-cold solution 3. The cell suspension was then gently layered on top of 12 ml ice-cold solution 3 and centrifuged at 300 ***g*** for 10 min. Neurosphere cultures were obtained by disposition of the supernatant and resuspension in neurosphere media [DMEM F12 Glutamax (Gibco) medium with 1×Neurobrew-21 (MACS Miltenyi Biotec), 1× penicillin/streptomycin (Gibco), 8 mM HEPES, 10 ng/ml EGF and 10 ng/ml FGF (Peprotech)] with cultivation at 37°C, 5% CO2. Cells were split after a dark spot became apparent in the middle of the spheres. Neurospheres were transferred into a 15 ml Falcon tube and centrifuged at 100 ***g***. The supernatant was discarded, the cells were resuspended in 1 ml Accutase (Millipore) and then incubated at 37°C for 5 min. To dissociate the neurospheres into single cells, cells were triturated several times with a Pasteur glass pipette and incubated again for 5 min. Cells were centrifuged for 5 min at 400 ***g*** and the Accutase was removed. Cells were washed twice in 5 ml PBS and centrifuged for 5 min at 400 ***g***. The cell pellet was resuspended in 1 ml neurosphere media and counted using a counting chamber. Cells were either passaged for expansion in flasks or 100,000 cells were seeded in PDL/laminin-coated cover slips in 24-well plates. The medium of seeded neurospheres was removed 1 day after seeding and medium without growth factors was added to the cells to trigger differentiation. On every third day the medium was changed. Cells were fixed at day 6 of differentiation. Proximity ligation assays were performed according to the manufacturer's instructions (Sigma Aldrich) using rabbit anti-BRN1 (a kind gift from Elisabeth Sock; Schreiber et al., 1997 1:500), rabbit anti-BRG1 (Santa Cruz Biotechnology, sc10768; 1:100), rabbit anti-FOXG1 (Abcam, ab18259; 1:500) and mouse monoclonal TCF4 (Santa Cruz Biotechnology, sc393407; 1:100)].

### DAPI staining at P7

Free-floating sections were washed twice with 1× PBS and incubated with DAPI for 10 min followed by another two times washing 1× PBS. Slides were mounted with 60 μl Mowiol and stored at 4°C. To quantify the length of the corpus callosum, the slices were counted from the first slice to last slice showing a corpus callosum. Brains were serially cut into 30 μm sections.

### ***In utero*** **electroporation**

Plasmid (pCAG-TCF4A-IRES-EGFP or tdTomato as control) was dissolved in saline to a concentration of 2 μg/μl with 0.05% Fast Green to monitor the injection. Pregnant ICR mice were anesthetized, and the uterine horns were exposed. Approximately 1 μl of DNA solution was injected into the lateral ventricle of embryos using a pulled glass micropipette. Each embryo within its uterus was placed between tweezer-type electrodes (CUY650P5), and 50 ms electronic pulses of 40 V were charged five times at 950 ms intervals using a square-pulse electroporator (CUY21SC; Nepa Gene Company). The uterine horns were placed back in the abdominal cavity, and the abdominal wall and skin were sewn with surgical sutures. Pups were harvested at P0.5 for immunohistochemistry. For each animal, matching slides were divided into 10 bins and the number of CTIP2^+^ cells and marker-positive cells was determined for each bin.

### ScRNA-Seq and analysis

#### Single-cell isolation of E18.5 cortex tissue

Neocortices of E18.5 embryos were dissected, and each cortex was incubated in 150 µl of Ovomucoid-Mix [1.15 mg/ml Trypsin-Inhibitor (Sigma-Aldrich), 0.53 mg/ml BSA, 400 ng/ml DNase I Type IV (Roche PVT) in L15 medium (Gibco)] and cut into small pieces. After addition of 150 µl of Papain-Mix [30 U/ml Papain (Sigma-Aldrich), 0.24 mg/ml cysteine (Sigma-Aldrich), 40 µg/ml DNase I Type IV (Roche PVT)], samples were incubated for 15-20 min at 37°C then 300 µl of Ovomucoid-Mix was added followed by a 5 min incubation at RT. The tissue was triturated with fire-polished glass pipettes, transferred to 10 ml of L15 medium and centrifuged for 5 min at 90 ***g***. About 9.5 ml of the supernatant was discarded. Cells were resuspended in the remaining media and strained (mesh size: 40 µm) to remove clumps. Cell density was determined using a Neubauer chamber. Libraries were prepared using the Chromium Controller and the Chromium Single Cell 3′ Reagent Kit v2 (10X Genomics) according to the manufacturer’s instructions. Single-cell suspensions were diluted in nuclease-free water according to the manufacturer's instructions to obtain a targeted cell count of 5000. Libraries were sequenced as described previously (Lukassen et al., 2018).

### Data processing for scRNA-Seq analysis using cell ranger and Seurat

The reads were de-multiplexed using Cell Ranger (version 2.1.1, 10X Genomics). mkfastq and read quality was assessed by FastQC (version 0.11.8, Babraham Bioinformatics). For mapping the reads to the mm10 genome (10X Reference 2.1.0, GRCm38, Ensembl 84) and to identify single cells, the standard Cell Ranger workflow was used. Common quality control measures for scRNA-Seq (gene count per cell, UMI count per cell, percentage of mitochondrial transcripts) were calculated using the Seurat R package (version 2.3.4) (Butler et al., 2018; Stuart et al., 2019). The analyses were performed for genotypes and for each mouse individually. Quality control thresholds were set to 1000-5000 genes per cells, 1800-10,000 UMIs and <6% of mitochondrial transcripts. Only samples with >500 cells after filtering were used to ensure a complete reproduction of cell diversity in the neocortex. Therefore, two samples for the WT and two samples for the KO were removed. Three samples for WT and two samples for KO were used for further analysis. We had to exclude one WT animal that displayed lower *Tcf4* expression than the KO and excluded cells that displayed a high background transcript expression of blood-related genes such as *Hbb-a1*.

### scRNA-Seq clustering and differential gene expression analysis using Seurat

Clustering of the cells was performed using the Seurat packages for R following the vignettes of the authors (Guided tutorial – 2700 PBMCs for PCA approach or Stimulated and Control PBMCs for multiCCA approach, version 2.3.4) (Butler et al., 2018; Stuart et al., 2019). Cluster identity was defined using known marker expression. To extract *Satb2*-expressing glutamatergic cells, the data were subset by accepting only cells belonging to the intermediate progenitor, newborn neuron, deep layer and upper layer clusters with counts for *Satb2* >0. DEGs between the two genotypes were determined using the MAST algorithm as implemented in Seurat with nUMIs as confounding variable to adjust for cellular detection rate (Finak et al., 2015). GO terms were identified with the Panther online tool (GO- Slim biological process and GO biological process complete) (http://www.pantherdb.org) (Mi et al., 2019).

### GRN analysis by SCENIC

Assessment of GRNs was performed using the R package SCENIC (version 1.1.01) (Aibar et al., 2017). Only genes expressed in at least three cells were considered for analysis, which was performed according to the package vignettes. After GRNs were defined, the networks were binarized. To that end, a threshold was set at the mean of the area under the curve that separated cells into cells with low and high activity. Cells clustered apart according to their genotype and differentially active GRNs were identified by hand. Only genes associated with the GO terms neurogenesis/neuron differentiation and with expression in at least one-quarter of the cells in the *Satb2* cluster were chosen for validation of the hypothesis that TCF4 interacts with the regulators. Common targets of TCF4 and SOX11 were found by intersecting the list of DEGs from the *Satb2* cluster with the predicted targets of the *Sox11* regulon. Enrichment for disease association in the list of differentially active regulons or of the common targets of TCF4 and *Sox11* was determined using DOSE (Yu et al., 2015).

### Statistical analysis

The Shapiro–Wilks test in R was used to determine normal distribution of all data. If normality could be assumed, the two-tailed Student's *t*-test of the ggplot2 implementation of R was used to determine statistical significance. Otherwise, a Mann–Whitney U test was performed using the ggplot2 implementation of R.

To determine whether differences in luciferase activities (Fig. 4E) were statistically significant, a two-tailed Student's *t*-test was performed using the ggplot2 implementation of R. Data are depicted as mean±s.d. (*P≤0.05; **P≤0.01; ***P≤0.001).

## Supplementary Material

Supplementary information

Reviewer comments
