# Supplementary material for: scRNA sequencing uncovers a TCF4-dependent transcription factor network regulating commissure development in mouse
Source: Development. 2021 Jul 19;148(14):dev196022. doi: 10.1242/dev.196022 (PMC8327186; doi:10.1242/dev.196022)
Supplement: Supplementary information [file develop-148-196022-s1.pdf]

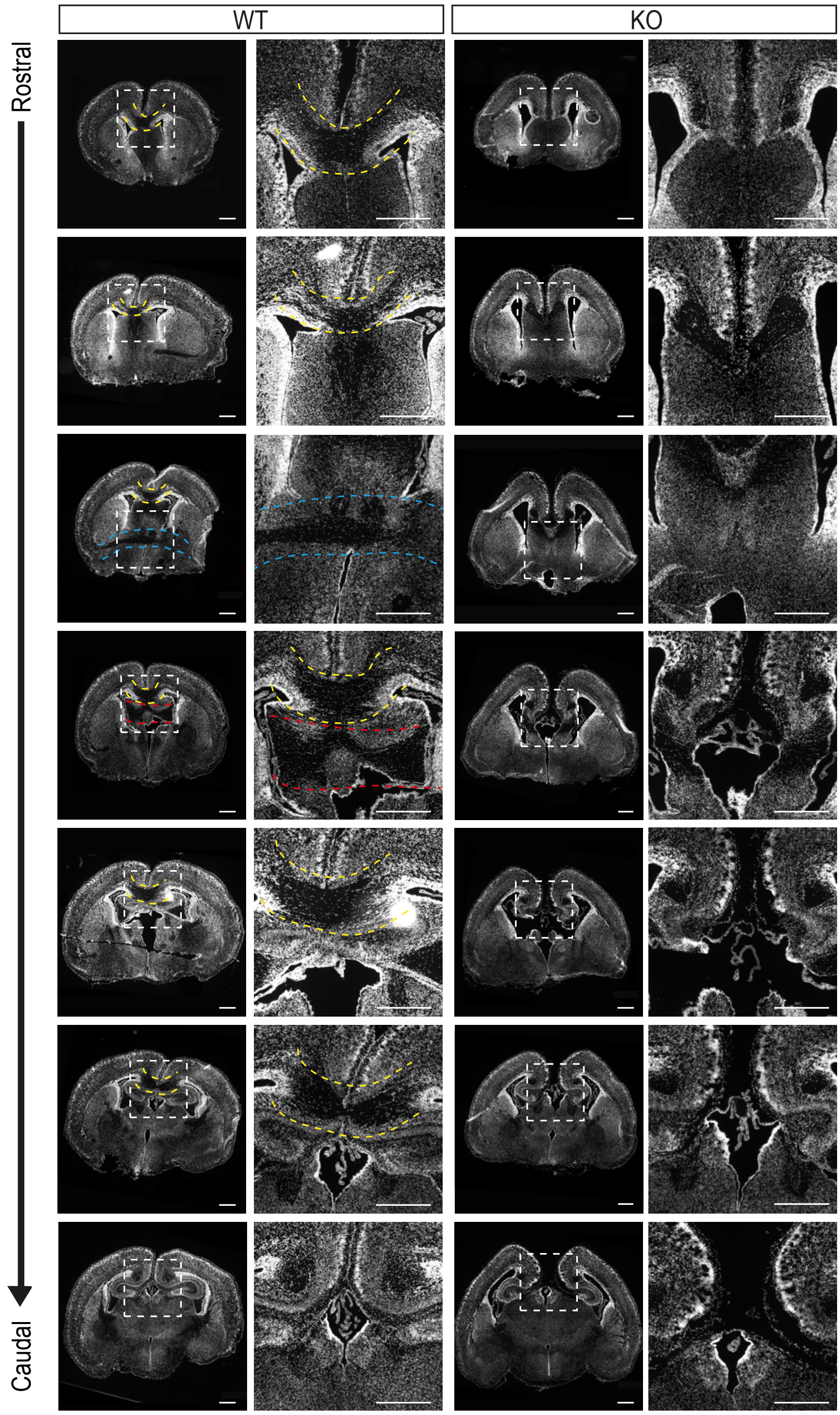

Figure S1. Overview images of the three forebrain commissures

Representative overview and magnification images of brain sections at P0 stained with DAPI showing the loss of the three commissure systems in *Tcf4KO* mice. Images on the right are magnification of the area marked with a with rectangle. Yellow dotted lines indicate the corpus callosum crossing the midline. Blue dotted lines indicate the anterior commissure and red dotted lines the hippocampal commissure. Scale bar, 500 µm.

Development • Supplementary information

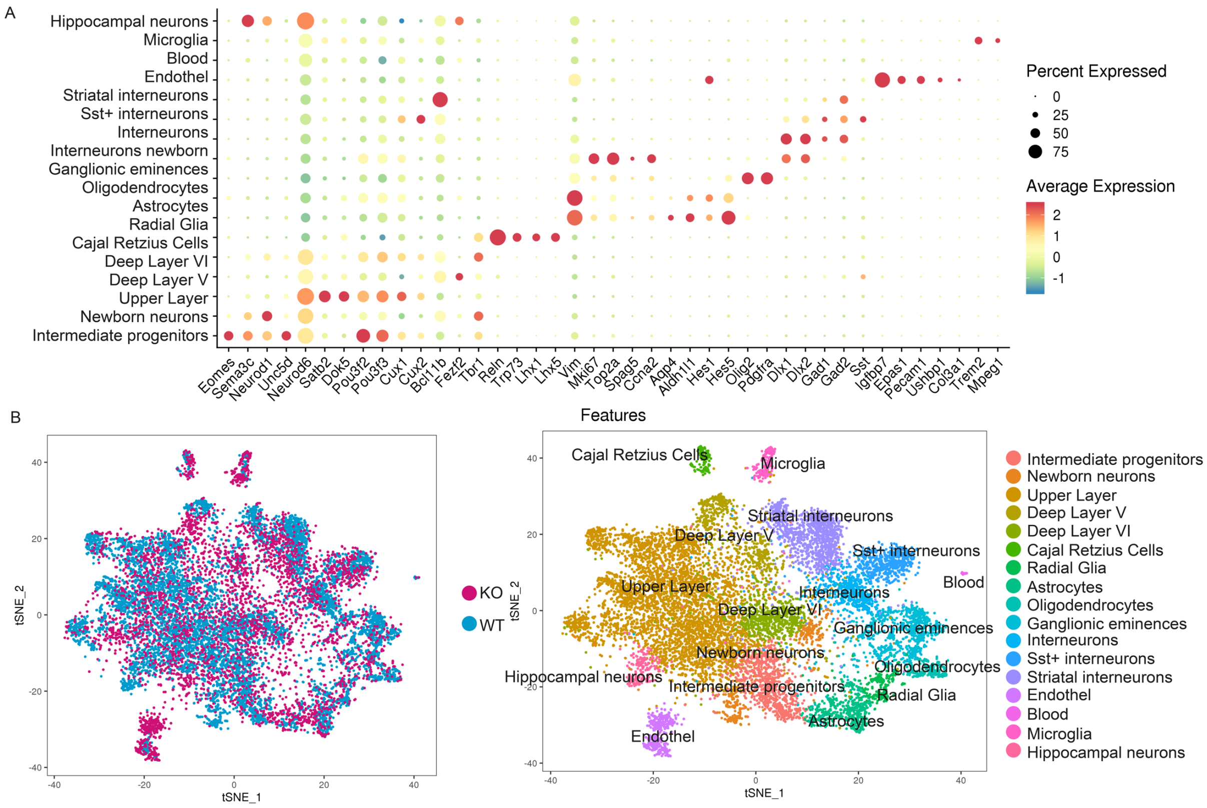

Figure S2. Clustering of the single cell dataset using the multiCCA approach

A Dot-Plot of cell clusters (y-axis) and representative marker used to assign the cell type (x-axis).

B tSNE-Plot coloured by genotype (left) and cluster identity (right).

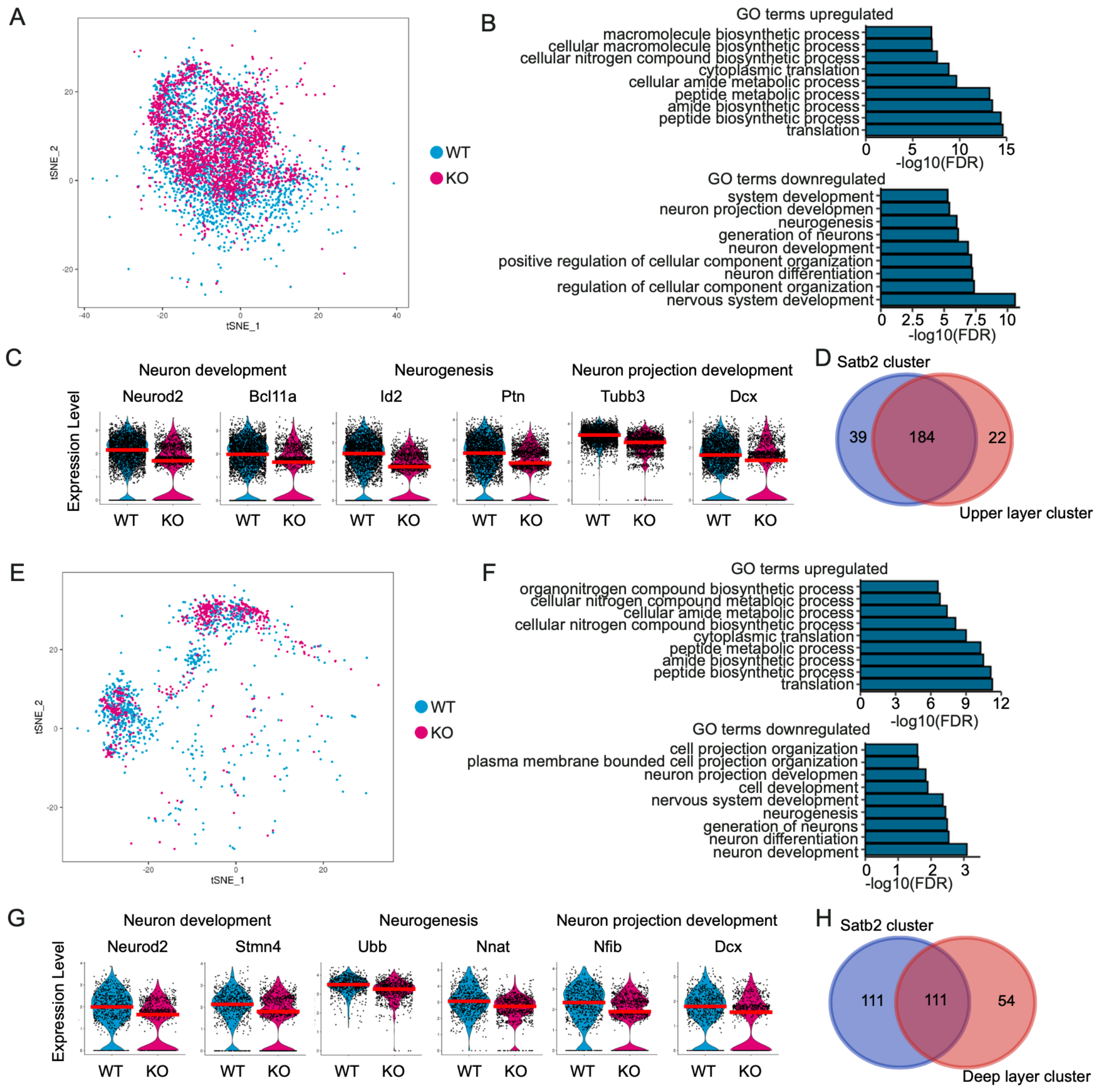

Figure S3. Differentially expressed gene analysis of the upper and deep layer cluster

A tSNE-Plot of the upper layer cluster used for further analysis.
B Selection out of the first 50 GO terms associated with up- and downregulated genes in the upper layer cluster. GO terms for neuron development, neurogenesis and neuron projection development were downregulated in the *Tcf4*KO cells.
C Violin Plots of differentially expressed genes in the upper layer cluster that are associated to neuron development, neurogenesis and neuron projection development. The red line depicts the median.
D Venn-Diagramm of the overlap of differentially expressed genes between the *Satb2* and UL cluster
E tSNE-Plot of deep layer cluster used for further analysis.
F Selection out of the first 50 GO terms associated with up- and downregulated genes in the deep layer cluster. GO terms for neuron development, neurogenesis and neuron projection development were downregulated in the *Tcf4*KO cells.
G Violin Plots of differentially expressed genes in the deep layer cluster that are associated to neuron development, neurogenesis and neuron projection development. The red line depicts the median.
H Venn-Diagramm of the overlap of differentially expressed genes between the *Satb2* and UL cluster

Development • Supplementary information

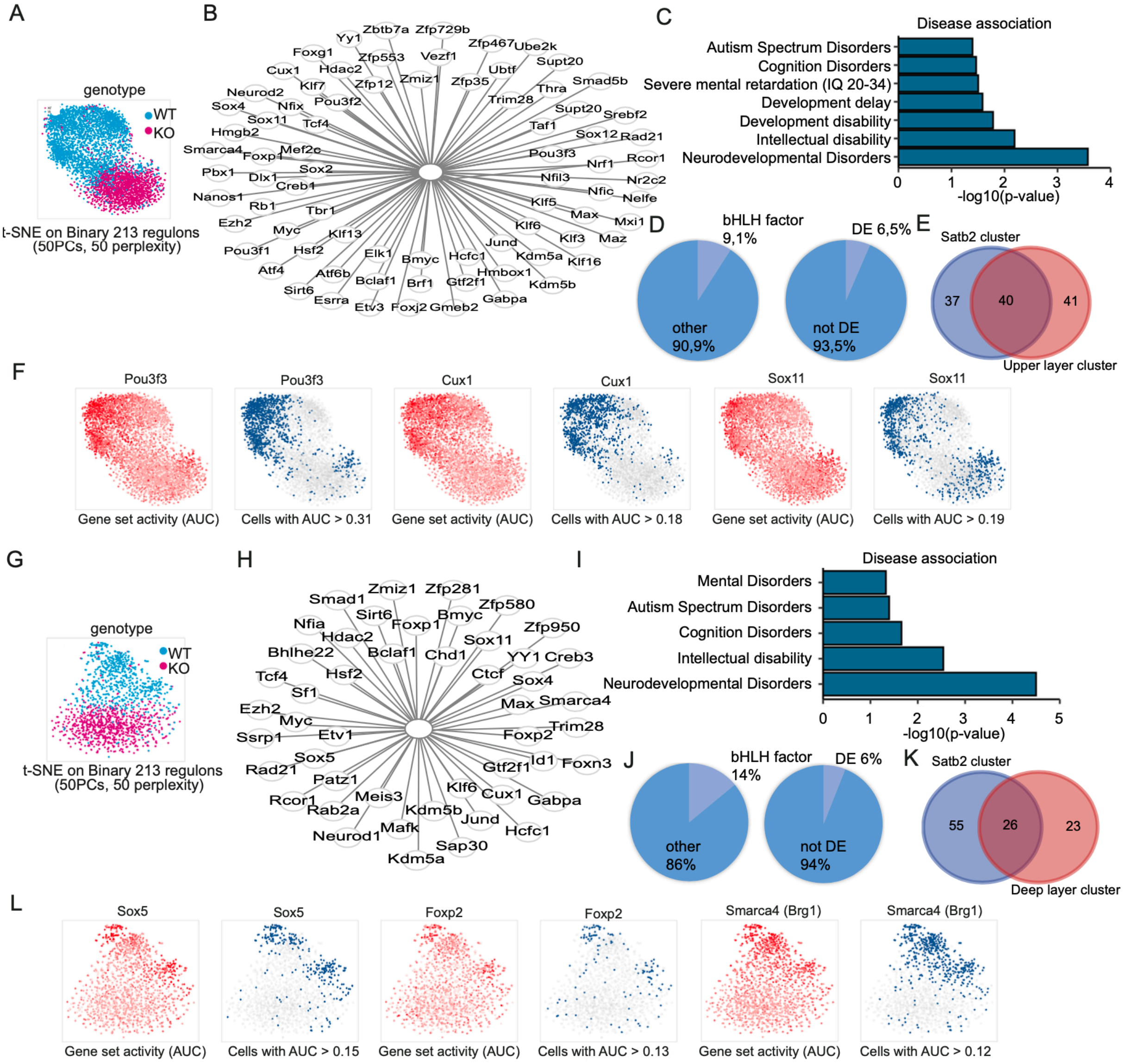

Figure S4. Gene regulatory network analyses of the upper and deep layer cluster

A tSNE-Plot of the upper layer cluster after GRN analysis. WT and KO cells segregated based on GRN activity with only minor overlap.

B Differentially active regulons of the upper layer cluster that may be possible interactors of TCF4.

C Selection of diseases association enriched in the list of differentially active regulons.

D Pie charts depicting the percentage of bHLH factors and differentially expressed regulators in the differentially active regulons.

E Venn-Diagramm of the overlap of differentially active regulons between the *Satb2* and UL cluster

F tSNE-Plots showing the regulon activity of *Pou3f3, Cux1* and *Sox11* in a continuous scale (left, red) or binarized (right, blue). The regulons are highly active in the WT cells with only a small number of KO cells showing a high expression.

G tSNE-Plot of the deep layer cluster after GRN analysis. WT and KO cells segregated based on GRN activity with only minor overlap.

H Differentially active regulons of the deep layer cluster that may be possible interactors of TCF4.

I Selection of diseases association enriched in the list of differentially active regulons.

J Pie charts depicting the percentage of bHLH factors and differentially expressed regulators in the differentially active regulons.

K Venn-Diagramm of the overlap of differentially active regulons between the *Satb2* and DL cluster

L tSNE-Plots showing the regulon activity of *Sox5, Foxp1* and *Smarca4* (also known as *Brg1*) in a continuous scale (left, red) or binarized (right, blue). The regulons are highly active in the WT cells with only a small number of KO cells showing a high expression.

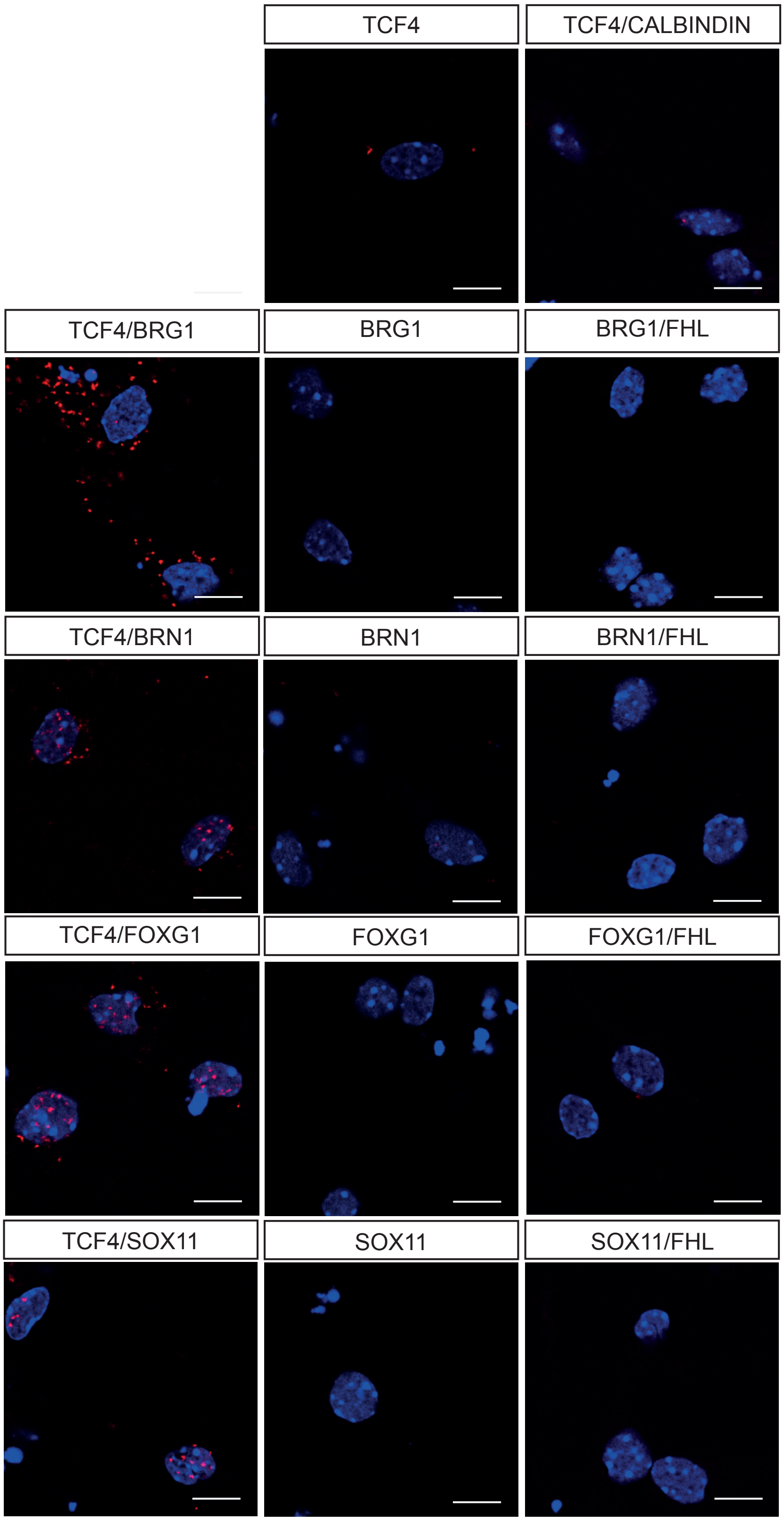

Figure S5. Proximity ligation assay in 6 days differentiated cortical neurospheres.

Left column: Proximity ligation assay using both mouse TCF4 and rabbit BRG1, BRN1, FOXG1 or SOX11 antibody. Middle column: Proximity ligation assay using only one antibody (ms TCF4, rb BRG1, rb BRN1, rb FOXG1 or rb SOX11) to control for unspecific amplification. Right column: Proximity ligation assay using mouse TCF4 and rabbit Calbindin or mouse FHL and rb BRG1, rb BRN1, rb FOXG1 or rb SOX11 to control for unspecific amplification. (n=3). ms = mouse; rb = rabbit.

Development • Supplementary information

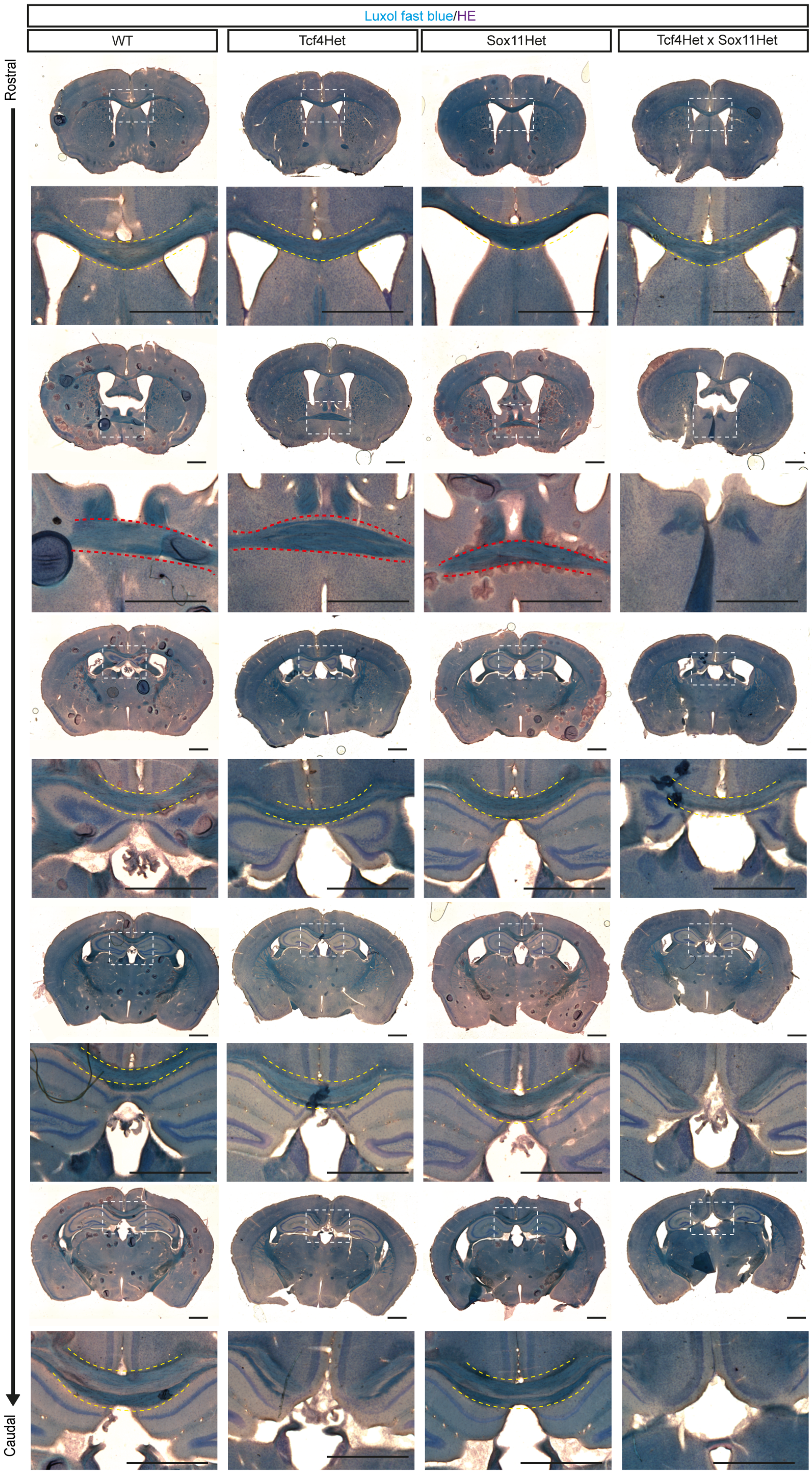

Figure S6. Overview images of Luxol fast blue stainings at P56

Representative overview and magnification images of Luxol fast blue stainings. Images below are magnification of the area marked with a white rectangle. Yellow dotted lines indicate the CC crossing the midline. Red dotted lines indicate the AC. In *Tcf4* and *Sox11* double haploinsufficient mice agenesis of the AC and agenesis of the splenium and caudal part of the body of the CC can be observed. Scale bar, 1000 µm, (n=5).

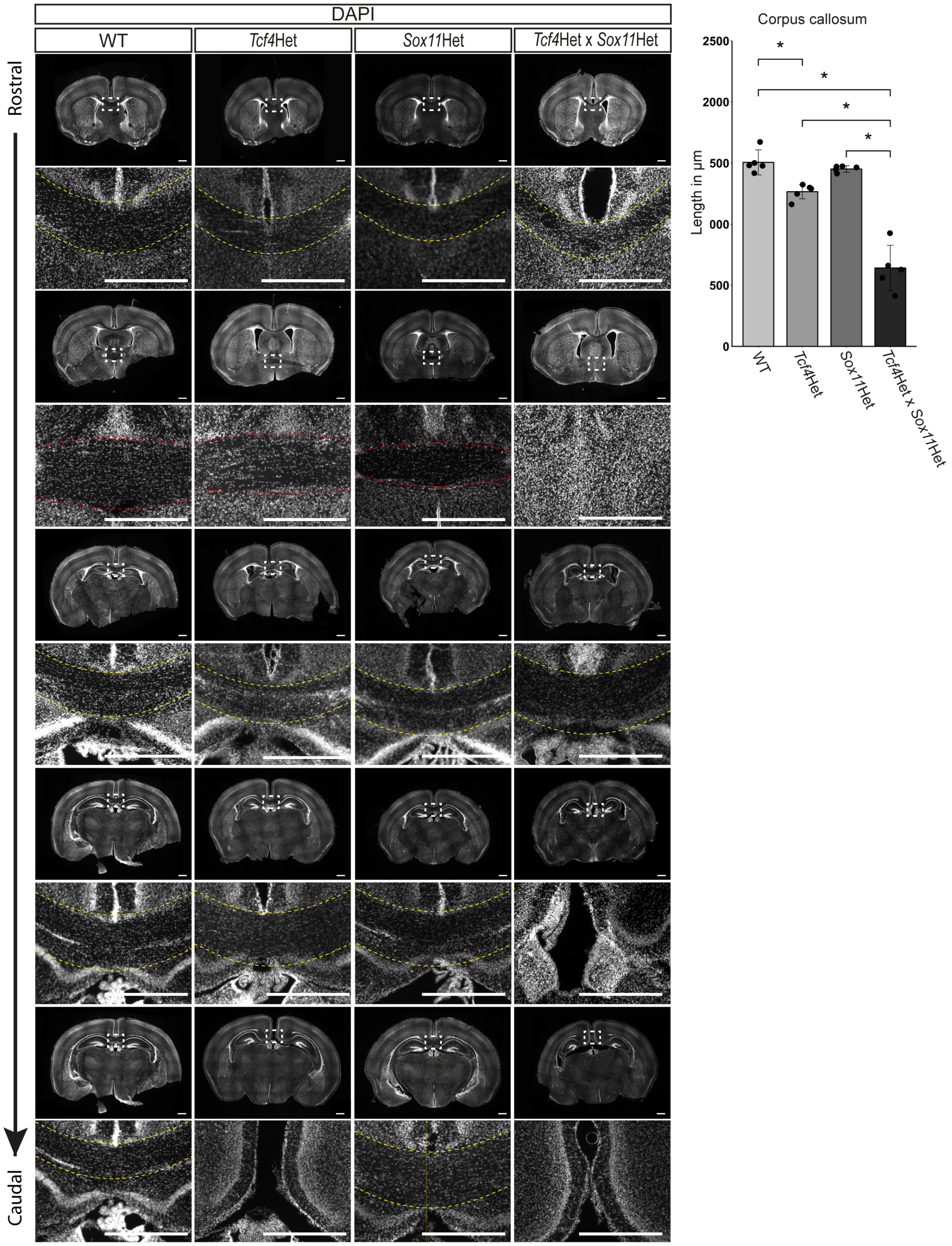

Figure S7. Overview images of DAPI stainings at P7

Representative overview and magnification images (DAPI) of brain sections at P7 showing the commissural system. Images below are magnification of the area marked with a white rectangle. Yellow dotted lines indicate the CC crossing the midline. Red dotted lines indicate the AC. In *Tcf4* and *Sox11* double haploinsufficient mice agenesis of the AC and agenesis of the splenium and caudal part of the body of the CC can be observed. Quantification of slices showing a corpus callosum is presented at the right. Scale bar, 1000 µm, (n=5, mean ± s.d, p-values were determined with Mann-Whitney-U test; WT 1506µm ± 91,78; *Tcf4*Het 1266 ± 51.61; Sox11Het 1452 ± 24; Tcf4Het x Sox11Het 642 ± 166,06; WT vs *Tcf4*Het: p-value = 0.012; WT vs *Tcf4*Het x *Sox11*Het: p-value 0.012; *Tcf4*Het vs *Tcf4*Het x *Sox11*Het: p-value = 0.012; *Sox11*Het vs *Tcf4*Het x *Sox11*Het: p-value = 0.011).

Development • Supplementary information

A Plxna2-ECR-Sequence:

CAGATTCTAAAGGTAAATCCCCAGAGAGGCATGCTTGAGGAAGTACTGAGGTGATTGCAGCACTGGACAG
CTGTGGGTTGGGCATGACACCTTCCAGACTCCAAGAGTAACCTGAGCAGAAGTTGAGGAGACAGGGGAT
CTTAGCTTTTTCCAGAGACAGAGGGTTGAAGGAGGAGGAGCTATGAGAAATGCAACAGGTACCAAGGGTA
GACCAAAATTATGAAAAGCATGAAAAGATGGCCTTTATCCCTCTAATGTTATGGCTATGTGGTGTTATTTTT
ATTCAAGGGAAGAAAAAAAAAAAAAAGAAAGAAAGAAAAGAATGCATGCTGCTCACCCAAACATCAGGACAT
TCTGTCCCTCTGTGATTGTGCTTCATGAAAATGCTTTCCAGCAAAAAATGAGAAAGAATGATTAAGGGGAA
AAAAATAACTAGAGCCACATTCATTTCAGCTTCTCTGAAAGCACAGCAGGAGGAAGTGGCTATGTTTGCCA
AACTCCAGAAAAAGACACAATTGACAGGAGGCGGGGAGGGGAGGGTGAGGCAGCAGAGGGCATATGGA
GGAGGGGTACCCCTTGTTTTATTCTCCCCTGAAGATACAGTAAATAATGAAGAGGTGTGGTGCACGGAGG
AGAAATTACTTAACAGGGCCCTTTCAGGGAGGAAAGTGGAACTAAACCCAGTCTCCATCATTTGTTCTGAC
ATCCAACTCCCTGTTTACATGGGCGATCCCTGGAGCTGACACTCCACTCTGCTGTCACAAGTAGTCCTTGA
CTGGAGCCGATCATCTCAAAATAATGCCCTGAAGTTCTTCCATTGTTGTGATTAACACAATGTAACATTGGC
CTCACTGACAACTGTGGATGGAGGAAGATAGCCTGCAGACCTCCCTGATTGTTGGAGCCAGAAAAAGTCC
CAATTAGTCCACTGGGTGGCACTCTCACTAGGGTTCTCTGCATCTTTTCTGGCCCATGGTGTCCACCAAAG
AATTGATTCAG

B DCX-Promotor-Sequence:

TGACTTCGTTTAAAAAACAACCAGTGTTGGATGCATGAGCCGAAATGTTAAAAAATTTACATATTTTTTATTT
TCTTTGAAGAAGATAAAAAGAGGAGATCTGTAATTTCTAAGAAACTTGATTTGGCCTGCTGAGTCCAGCCA
CTAGGCAGAAGGTTTTAGCCAAGTAAAATTGCCAATTTTCTAAGAGAAAGGGCTAGCACATTGCTCATTAG
AGCATTCTGAGCTTGCCTGTGCAATCTTTTTTTTCCTACCCTGCAATTTCCTGTGCGTTATAAACGAAACCT
TTCTAGCTGTTAATGCAGGCTGTGAATTGAAGAAAAAAAAGCATGTAATTAATCATAGGAGGTTGGGGGTG
TTCGCTAAGCTTCAGTTACAGGGGAGAAGCTGGACAAGGCACTAGGACCTAGAAGGCAACTATCCACCCT
GGCAGGAATTTCTTGCTTGGAGCTCAGACAACAAAGGCATAGAGAGATTGGTTTCTTTCTCTCAGCATCT
CCACCCAACCAGCAGAAAACCGGTGAGTGGGGCTTTCGAGTGATTTTCAAGCAGAATGTAACAGATGTCA
ACCGGGAAAGCACAAGGCACACGGCTTTCTTTCTCTGTGTGTTCGCCTCTTTCTTCTCTTTTATTTGCCTTA
TTCTATAGGATTTTTGTCCTCTAAGATTCTACCTGGGATTTTCCTTTGGAAAAGTGAGTTTGTTGTTCCTTTG
TTTTCACTATGATGCTAATTTAGAATAATAGCACTTCTGATTCTAAAGCATAGCTTTATTTGCACAGCCTGCC
TGGGGAAAATGCTTGCTACTCATCTTGAGGAGGTGGGCTCTTACTACTGCAGGTTGTCTGACAGAGACAA
TGCTGAGCTCAGCATAGGTCATGGTGACACTGGAAAAAAAGGGGGTACTGAGCCTGGCAAATATACCAAC
TACCAGTCCTCCTTTATCTCCTTTCTCCCTGGTTTCTTGCAAATCTCGATGTGGCAGTATATATATATAGCAGC
TGAGCCCTCTTGCTTTGTGAGTCTTTTTCCCCCATTTGTGAGATGAATGTTAATAGTTTGGTTTCTTGGAT
GTCACATTACCTTTGTAAGGGGTTAGGGCTTTGGTTGTATTATTGGGTTGCATGTTTTCATTGTTTTGGACG
TTTTTTTTTCTGGTGGGGGACGGGTTCAGGGGGGTTGAAATCCAAGCTTGACAGATGACTTTTTTTTTCCC
TCCATCAATACACCTAAGCAATAGACAAGTTTGAAGTGAATTGCCTGCTTCGAGGGCAAAATATTCCTTCA
GTCAGGGGAGAAACCCAGAACAATGAAAGGTGTACCTACTTGGAAAGGTCCCATGTGCTATTCAGGGACC
CATTTGGGAATCTTTCCACAATTATTCCATTAAGAGGTGTTGCTGCATTCATTGGTCGGGGAGGGGATGAA
ACACCTGAAAGGAGAAAAAGGATTCTGTGATCAAATGGAAATGAAAGGGAAGCAGAGCTAATAGCTTGCT
AAATAACTGGGTTTTTTCGACAATCCCTCCCCCTTTTAGACCCCAGCTTATTTCTTATGGATGCCGTATAGC
GGCACCAGCTTGATGGGGAGAGGGTTTGATGAATAGCACAAAGGCACTGGGTATTCCCTGGAGGCTGTC
CCTTTAAAAGAGAATCCTAGTTTATTCTGGGGGAGGGGATACACATATTAGAGCAGGCAAAAAAGGACAAG
GAATAAAAGTAATTCACCCCCTTCCTAGCCATTGTATTGAGATGCAAAGGCTGCTTCCTACAGGAGGGTGC
TAACCTTGGCTAGCTCCCTCTGTTTCTCTTTGAGGGAATTTAGTCAGGCTATGGATTCATTTACAACTGTTA
GTCATGTGGCCATGTGTGAAGGAGCAGATGCCAGTTTTAATGTATTTTGCCCGAAGTTACAATTTGATAGG
AGCCACTGTCAGGAAGCTCCAGGTTTTTAAGCTATTTCAACACGCCCTCCCCAAATTGGAACAGTGCCAAA
AGTGCCACCCTTTCTATCTCTTCCTCCTATCCCCCTCCCCACCATTCAGTCCTCAGCCTACTGCCCAGCCC
CCTCCTTCTTCTCTATTAAGATCAATATTCCTGCAGGTCAGGGACAAGCAGCAGATGGGTCACAGGCTTTT
TTCAACCAGTTCTTTTCACAGGCAGCAGATTGCAGCTCTGGATCTGGCTAATATTT

Figure S8. Evolutionary conserved regions for *Plxna2* and *Dcx* used in luciferase constructs

A ECR of *Plxna2*; Chr1: 194,607,138-194,608,136
B Promotor region of *Dcx*; ChrX: 143,931,400-143,933,590
The letters written in red indicate putative Sox11 binding sites, the letters written in blue indicate E-Boxes (putative TCF4 binding sites). Highligthed in yellow are the sites of the oligonucleotides used in EMSAs.

Development • Supplementary information

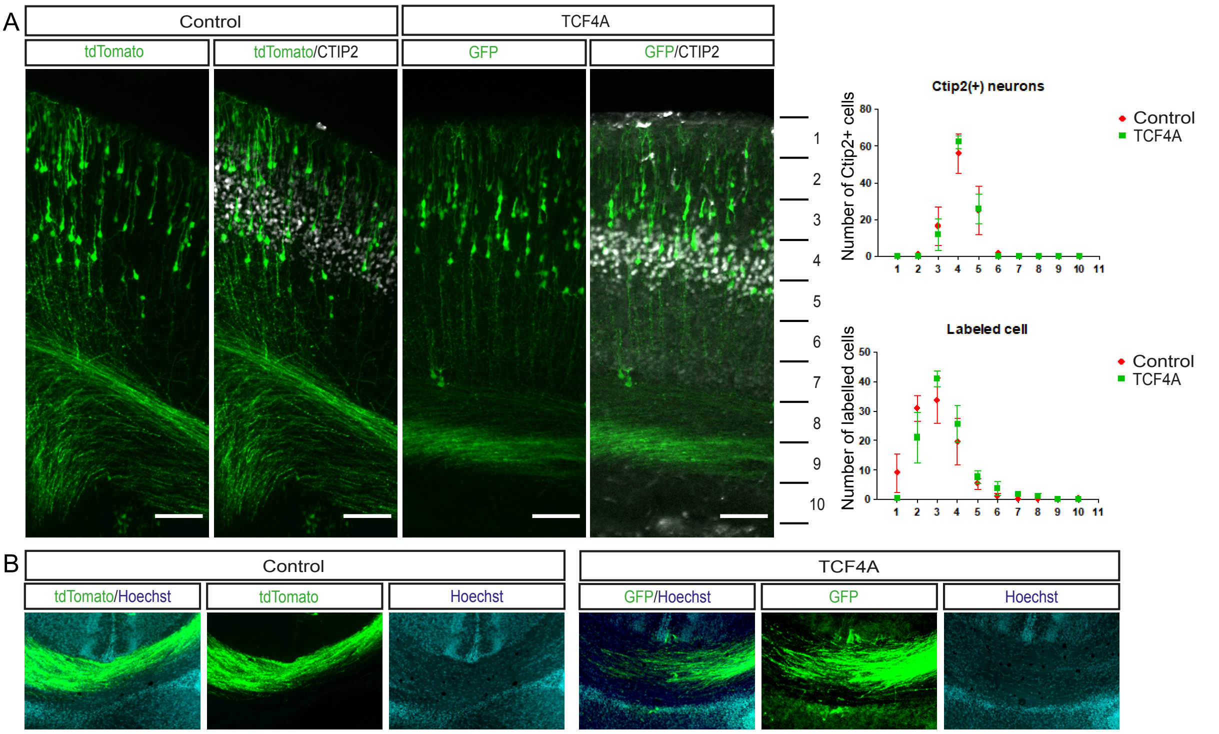

Figure S9. Analysis of TCF4A overexpression by *in utero* electroporation and the binding of TCF4A

A Expression constructs were introduced into E13.5 wild-type brains through *in utero* electroporation. Brains were dissected out at P0.5 for analyses. Cells that had taken up the expression construct are detected by the expression of the fluorescent reporter (coloured in green). Quantification of control pCAG-tdTomato vector and pCAG-TCF4A-IRES-GFP vector electroporated cells and expression of CTIP2 (grey) in the cortical plate. No difference in the number of CTIP2+ cells or the distribution of labelled cells was observed. n = 3, scale bar = 100 µm
B Expression vectors were *in utero* electroporated into E14.5 wild-type brains. Brains were dissected out at for analyses. Electroporated cells are shown in green. Note that processes of TCF4A overexpressing neurons are able to cross the midline. n = 3, scale bar = 100 µm

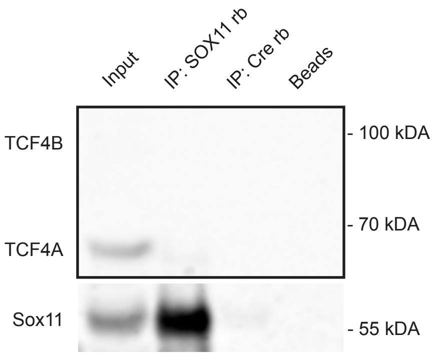

Figure S10. Analysis of TCF4A binding to SOX11 using TCF4KO protein lysates

Co-immunoprecipitation assay conducted with E18.5 cortex lysates from *Tcf4*KO mice using anti-SOX11 antibody. Upper panel: detection with anti-TCF4 antibody. Lower panel: detection with anti-SOX11 antibody. The blots presented are cropped. TCF4B was co-immunoprecipitated with SOX11, but not with an isotype control for IgG and Agarose A Beads alone. The interaction was confirmed in three independent biological replicates (n = 3); rb = antibody raised in rabbit.

Table S1. Differential expressed genes in the *Satb2* cluster and GO term analysis. Related to Figure 3.

Click here to Download Table S1

Table S2. Differential expressed genes in the limited *Satb2* cluster and GO term analysis. Related to Figure 3.

Click here to Download Table S2

Table S3. Differential expressed genes in the upper layer cluster and GO term analysis. Related to Supplemental Figure 3.

Click here to Download Table S3

Table S4. Differential expressed genes in the deep layer cluster and GO term analysis. Related to Supplemental Figure 4.

Click here to Download Table S4

Table S5. Differential active regulons in the *Satb2*, the limited Satb2, the deep layer and the upper cluster. Related to Figure 3 and Supplemental Figure 3 and 4.

Click here to Download Table S5

Table S6. Overlap of differential expressed genes and the predicted Sox11 regulon in the *Satb2* cluster and GO term analysis. Related to Figure 5.

Click here to Download Table S6
