# Supplementary material for: scRNA sequencing uncovers a TCF4-dependent transcription factor network regulating commissure development in mouse
Source: Development. 2021 Jul 19;148(14):dev196022. doi: 10.1242/dev.196022 (PMC8327186; doi:10.1242/dev.196022)
Supplement: Reviewer comments [file develop-148-196022_review_history.pdf]

scRNA-Sequencing uncovers a TCF4-dependent transcription factor
network regulating commissure development in mouse

Marie-Theres Wittmann, Sayako Katada, Elisabeth Sock, Philipp Kirchner, Arif B Ekici, Michael Wegner, Kinichi Nakashima, Dieter Chichung Lie and André Reis
DOI: 10.1242/dev.196022

Editor: Paola Arlotta
Review timeline

Original submission:	19 August 2020
Editorial decision:	19 October 2020
First revision received:	29 April 2021
Editorial decision:	24 May 2021
Second revision received:	1 June 2021
Accepted:	15 June 2021

Original submission

First decision letter

MS ID#: DEVELOP/2020/196022

MS TITLE: scRNA-Sequencing uncovers a TCF4-dependent transcription factor network regulating commissure development

AUTHORS: Marie-Theres Wittmann, Philipp Kirchner, Arif B Ekici, Elisabeth Sock, Dieter Chichung Lie, and André Reis

I have now received all the referees' reports on the above manuscript, and have reached a decision. The referees' comments are appended below, or you can access them online: please go to BenchPress and click on the 'Manuscripts with Decisions' queue in the Author Area.

As you will see, the referees express considerable interest in your work, but have some significant criticisms and recommend a substantial revision of your manuscript before we can consider publication. If you are able to revise the manuscript along the lines suggested, which may involve further experiments, I will be happy receive a revised version of the manuscript. Your revised paper will be re-reviewed by one or more of the original referees, and acceptance of your manuscript will depend on your addressing satisfactorily the reviewers' major concerns. Please also note that Development will normally permit only one round of major revision.

We are aware that you may be experiencing disruption to the normal running of your lab that make experimental revisions challenging. If it would be helpful, we encourage you to contact us to discuss your revision in greater detail. Please send us a point-by-point response indicating where you are able to address concerns raised (either experimentally or by changes to the text) and where you will not be able to do so within the normal timeframe of a revision. We will then provide further guidance. Please also note that we are happy to extend revision timeframes as necessary.

Please attend to all of the reviewers' comments and ensure that you clearly highlight all changes made in the revised manuscript. Please avoid using 'Tracked changes' in Word files as these are lost in PDF conversion. I should be grateful if you would also provide a point-by-point response detailing

© 2021. Published by The Company of Biologists under the terms of the Creative Commons Attribution License (https://creativecommons.org/licenses/by/4.0/).

how you have dealt with the points raised by the reviewers in the 'Response to Reviewers' box. If you do not agree with any of their criticisms or suggestions please explain clearly why this is so.

Reviewer 1

Advance summary and potential significance to field

In their manuscript Wittmann et al. addressed the function of TCF4 during cortical development. They confirm recent findings that deletion of the long variant of TCF is lethal and results in cortical agenesis.
They show that one effect of TCF4 mutation is an increase in SATB2 expression upper layer cortical neurons at the expense of CTIP2 expressing neurons. They show that although SATB2 expressing putative commissural neurons are generated in excess, the TCF mutant mice show an agenesis of most cortical commissural tracts. They then performed scRNA-seq in order to try to identify the defect with a major focus on the SATB2 expressing neurons in the mutant compared to control. They identify transcriptional networks and transcription factor regulons that are miss-regulated in the TCF4 mutant neurons. They show TCF4-Sox11 cross-interactions and identify putative regulatory elements that, at least in heterologous cell systems, show a synergistic regulation by TCF4 and Sox11. It is unfortunate that a recent paper describing the TCF4 mutant mice and some of the cortical defects reduces the main impact of the phenotypes described but the authors attempt to go further to address the molecular mechanisms of the TCF4 mutation. The authors potentially have interesting findings that could have an impact not only on how we interpret development of the cerebral cortex but also for human neurodevelopmental disorders.
However, the data are somewhat speculative at present and need experimental support and a clear description of whether they authors think the TCF4-Sox11 axis is a major contributor to the cortical agenesis they see.

Comments for the author

Main concerns
1. It is unfortunate that the mutant that the authors analyze is not a complete knockout and results in expression of a short, endogenous form of TCF4. The authors need to show if this short form has a function in the absence of the longer TCF4 isoform. A better description of the long and short forms of TCF4 and a comparison of the relative domains would help. Addressing the effects of overexpressing the short form of TCF4 in cortical progenitors in vivo, potentially by in utero electroporation or viral expression and analysis of CTIP2 and SATB2 neurons may be able to circumvent any doubt about dominant effects over the longer TCF4 protein.

2. The images in Figure 1D are too small to see what the authors want to show with axonal midline crossing. They should add higher magnification images to the main figure.

3. Figure 1F and H. This reviewer cannot workout what the authors want to show. Either higher magnifications are needed and/or some arrows and indicators are needed on the panels.

4. Does the short form of TCF4 interact with Sox11 or affect the binding of the long form of TCF4 to Sox11?

5. The authors show that Sox11 and TCF4 can cooperate on a putative regulatory elements in the Dcx and Plxna2 genes. The authors need to describe the locations of these regulatory elements within the genes relative to TSS. They should also show the sequence of the putative regulator elements examined in their Luc assays. In addition, a better description of the element used in the reporter assays is needed.

6. Heterologous overexpression reporter assays are notorious for giving false results. The authors need to show whether the Sox11-TCF4 regulation of the Dcx and Plxna2 elements occurs in vivo. Some evidence of Sox11 and TCF4 interaction with these elements in cortical neurons is needed. ChIP PCR over the elements with Sox11 and TFC4 would not be direct proof of co-occupancy but would lend strong support.

© 2021. Published by The Company of Biologists under the terms of the Creative Commons Attribution License (https://creativecommons.org/licenses/by/4.0/).

7. Sox11 can regulate both the Dcx and Plxna2 elements in vitro. The authors identified these two genes as miss-regulated but is Sox11 expression changed? As the authors claim that Sox11 can induce these elements alone, the authors need to show or at least discuss why this is not happening in vivo and if Sox11 is regulating them in vivo in the absence of TCF4, why these findings are relevant.

8. It is clear unclear whether the authors think that Dcx and Plxna2 are contributing significantly to the TCF4 mutant phenotype. Radial migration does not seem to be affected which would be expected if Dcx is affected in the mutants. The authors should discuss and comment on this.

9. The authors see an increase in SATB2 at the expense of CTIP2 expressing neurons. CTIP2 and SATB2 are known to be in a cross-regulatory network and repress each others expression during fate acquisition.
The authors focus on SATB2 expressing cells and show that some of these are actually misplaced in the deeper cortical layers where CTIP2 neurons are expected. Does TCF4 regulate CTIP2 expression? Are the authors sure they are not comparing a mixed population of neurons in the mutant with SATB2 neurons in the control. If CTIP is downregulated in the mutant neurons it is to be expected that SATB2 is not repressed and thus deep layer neurons are retaining SATB2 expression? This would confuse the scRNA-seq analysis. The authors should not use small numbers of marker genes and RNA expression to define cell types.

10. The authors should also compare the CTIP2 neuron populations in the mutant and the controls and extend the marker panel to examine whether they are including CTIP2 neurons in their SATB2 mutant populations.

Minor comments
11. What is the variance covered by tSNE1 and tSNE2 in the analyses?

12. It is surprising that the authors identify a significant number of GE-derived neurons in their sequencing. Is this due to contamination, the result of GE derived interneurons migrating dorsally, or a miss annotation? The authors should comment.

13. The coIP data are not convincing. The blots are not of good enough quality particularly for the over expression Ips in Figure 3G. The lanes are all different sizes due to running and buffer artefacts. The authors should also include in the legend of Figure 3G and 3H that ms and rb refer to the species in which the primary antibody was raised.

Reviewer 2

Advance summary and potential significance to field

This manuscript identifies novel binding partners for the TCF4 transcriptional regulator derived from gene regulatory network analysis based on single cell transcriptomics on loss-of-function mice. The described pipeline appears to yield verifiable candidate interactors as demonstrated by the investigation of TCF4-Sox11 interactions using co-immunoprecipitations and luciferase assays. However, I would note that although the compound TCF4/Sox11 mutant exhibits a similar phenotype, this does not offer conclusive evidence that TCF4 and Sox11 directly interact in vivo. Instead, it only indicates that these proteins act in the same pathway.
The network analysis has identified a panel of other potential TCF4 cofactors, thereby providing the starting point for further exploration of TCF4 function.

Comments for the author

Although the transcriptomics is informative, the analysis of the mutant phenotypes is superficial and the images of the stained sections are very poor (Figs 1C,H and 4A). It is not clear what histochemical method was used to stain the sections in Fig 1C and the commissures are barely visible in the wildtype cortices (Figs 1C, 4A). The sections used for the wildtype and mutant appear to be poorly matched (Figs 1C,D,H and 4A), making it difficult to directly compare sections. This

© 2021. Published by The Company of Biologists under the terms of the Creative Commons Attribution License (https://creativecommons.org/licenses/by/4.0/).

will directly impact the results when quantify neuronal subtypes. The cortical thickness of the mutant (Fig. 1C and D) appears to be reduced in the mutant, however this is not taken into account when quantifying the neuronal subpopulations. Note that Mesman et al (2020) report that TCF4 mutants exhibited a reduced cortical plate. We are not given any information as to how the quantification (Fig 1E) was performed and normalized.

In Fig.1D the mutant cortices appear smaller and quite distorted. A thorough description of the cortical cytoarchitecture is lacking, making it difficult to interpret the phenotype. DiI tracing indicates that CC axons are delayed in crossing the midline. This could be due to the aberrant cortical architecture, ie. a non-cell autonomous effect. Thus, it is possible that axonal growth is only delayed or inhibited due to the local environment? This possibility should be investigated, as if true, the role of TCF4 would need to be revaluated.

An in vitro assay would address this question.

The phenotype of the TCF4 mutants as described here appears to be distinct from that reported by Mesman et al (2020). In contrast to this manuscript, Mesman et al report an absence of midline remodelling and an increase in Ctip2+/Satb2+ neurons in layers V/VI. This should be discussed in the Discussion section.

However, these differences may be resolved if a more thorough analysis is performed.
Analysis of the axon tracts of the compound TCF4/Sox11 mutants is performed at p56 only. How does the phenotype of these mutants compare with the TCF4 mutants embryonically?
There is no description of the single cell FACS sorting protocol in the Materials and Methods.
Were 3 independent biological replicates performed for the luciferase assays?
The proximity ligation assays have not been controlled appropriately. Irrelevant isotype-matched control antibodies should be paired with the specific antibodies.
Some references are incomplete.

First revision

Author response to reviewers' comments

Response to Comments:
Reviewer 1 Comments for the Author:
Main concerns

1. It is unfortunate that the mutant that the authors analyze is not a complete knockout and results in expression of a short, endogenous form of TCF4. The authors need to show if this short form has a function in the absence of the longer TCF4 isoform. A better description of the long and short forms of TCF4 and a comparison of the relative domains would help. Addressing the effects of overexpressing the short form of TCF4 in cortical progenitors in vivo, potentially by in utero electroporation or viral expression and analysis of CTIP2 and SATB2 neurons may be able to circumvent any doubt about dominant effects over the longer TCF4 protein.

Answer: We have followed the reviewers suggestion and included a more detailed description of the long and short forms of TCF4. As suggested by this reviewer, we have performed in utero electroporation in E13.5 and E14.5 wildtype mice to evaluate potential dominant effects of the short TCF4 isoform over the longer TCF4 isoform (i.e., TCF4B). Overexpression of the short TCF4A isoform did not affect cortical layering. Moreover, TCF4A overexpressing neurons were still able to send interhemispheric projections across the corpus callosum. These data indicate that the short TCF4A isoform does not have dominant effects over the longer TCF4 isoform.
We would also like to point out that mouse models with a knockout in the DNA-binding bHLH domain, which affects all isoforms of TCF4, show a phenotype (loss of commissures) that is highly similar to the phenotype of the TCF4 mutant mice that were analyzed in our study (Mesman et al.,

© 2021. Published by The Company of Biologists under the terms of the Creative Commons Attribution License (https://creativecommons.org/licenses/by/4.0/).

2020). The similarity in phenotype indicates that expression of the long isoform of TCF4 is critical for commissure development and that expression of shorter TCF4 isoforms does neither compensates nor aggravate the phenotype

The data are presented now in Figure S10 and discussed in the discussion section and read as follows: Line 282-296:

The present knockout model allows for the expression of shorter TCF4 isoforms with an intact bHLH DNA binding domain such as TCF4A. Reporter assays of genes that were predicted to be regulated by cooperation of TCF4 with SOX11 indicated i) that cooperativity with SOX11 was specific to TCF4B, and ii) that TCF4A did not impair SOX11-dependent transcriptional activity. Co-IP analysis of the E18.5 cortex also indicated in vivo biochemical interaction of TCF4B with SOX11 but not of TCF4A and SOX11. Furthermore, in utero overexpression of TCF4A at E13.5, had no adverse impact on cortical layer generation (Fig. S10A) and TCF4A overexpressing neurons were capable of crossing the midline (Fig. S10B). These data indicate that the cortical layering phenotype and loss of intercortical projections is caused by the loss of the longest TCF4 isoform and not by the residual expression of TCF4A. Together with the similarity in phenotypes between the present knockout mice and the Tcf4KO mice generated by disruption of the bHLH domain, these findings indicate that the remaining expression of shorter isoforms such as TCF4A has negligible impact on the loss-of-TCF4B commissural phenotype.

2. The images in Figure 1D are too small to see what the authors want to show with axonal midline crossing. They should add higher magnification images to the main figure.
Answer: We have now added higher magnification images to the figure and added Supplemental Figure S2 for more details.

3. Figure 1F and H. This reviewer cannot workout what the authors want to show. Either higher magnifications are needed and/or some arrows and indicators are needed on the panels.

Answer: Thank you for this comment. We have rearranged the previous figure 1 and split it into two in order to provide higher magnification images to better illustrate the analysis of the commissural phenotype. We have also added indicators to the panels. The previous panels 1 F-H are now panels 2 B- D.

4. Does the short form of TCF4 interact with Sox11 or affect the binding of the long form of TCF4 to Sox11?

Answer: Thank you for this comment. We detect the long and the short isoform in cortex lysate from E18.5. In the co-immunoprecipiation analysis we, however, only detect interaction between the long isoform of TCF4 and Sox11 (Figure 4H). We therefore suggest that Sox11 predominantly interacts with the long isoform of TCF4. This finding is described in lines 199-205, which read as follows:
Lines 199-204: We sought to genetically validate the functional relevance of the interaction of TCF4 and SOX11 in commissural development in vivo. Co-IPs from E18.5 neocortex lysates confirmed the interaction of TCF4B with SOX11 in vivo (Fig. 4H). Notably, we did not observe a binding of SOX11 to TCF4A in the in vivo Co-IPs although TCF4A was detectable in the input (Fig. 4H), suggesting that robust interaction of TCF4 isoforms with SOX11 depends on domains encoded within the first four exons of the Tcf4 gene.

5. The authors show that Sox11 and TCF4 can cooperate on a putative regulatory elements in the Dcx and Plxna2 genes. The authors need to describe the locations of these regulatory elements within the genes relative to TSS. They should also show the sequence of the putative regulator elements examined in their Luc assays. In addition, a better description of the element used in the reporter assays is needed.

Answer: We agree with this reviewer that it would be highly to provide information on the putative regulatory elements in the DCX and Plxna2 genes. The information is now provided in Figure 5, Supplemental Figure 9 and the Material and Methods section.

© 2021. Published by The Company of Biologists under the terms of the Creative Commons Attribution License (https://creativecommons.org/licenses/by/4.0/).

6. Heterologous overexpression reporter assays are notorious for giving false results. The authors need to show whether the Sox11-TCF4 regulation of the Dcx and Plxna2 elements occurs in vivo. Some evidence of Sox11 and TCF4 interaction with these elements in cortical neurons is needed. ChIP PCR over the elements with Sox11 and TFC4 would not be direct proof of co-occupancy but would lend strong support.

Answer: The reviewer is correct to point out that overexpression reporter assays can give false results. As suggested by the reviewer we tried to establish a ChIP-PCR protocol from E18.5 cortical tissue, however, we found currently available antibodies for SOX11 and TCF4 to be unsuited for ChIP-PCR and were not able to establish a reliable protocol. We therefore performed EMSAs with oligonucleotides containing possible binding sites for TCF4 and SOX11 in the identified regulatory regions of Plxna2 and Dcx (Supplemental Figure 9) and were able to show that both TCF4 and SOX11 directly bind to the regulatory elements which is highly suggestive for a direct interaction of these transcription factors with the respective regulatory elements (Figure 5G).

7. Sox11 can regulate both the Dcx and Plxna2 elements in vitro. The authors identified these two genes as miss-regulated but is Sox11 expression changed? As the authors claim that Sox11 can induce these elements alone, the authors need to show or at least discuss why this is not happening in vivo and if Sox11 is regulating them in vivo in the absence of TCF4, why these findings are relevant.

Answer: As the reviewer correctly points out, Sox11 by itself is able to stimulate the activity of Dcx and Plxna2 elements in vitro. Sox proteins typically show gain in activity by interaction with other transcriptional regulators (Kamachi et al., 2000, Reiprich and Wegner, 2015), which could explain our finding that while Sox11 expression is unaltered, expression of its targets such as Dcx and Plxna2 is reduced. We are discussing this point in the revised manuscript in line 241-249, which reads as follows:
TCF4A or TCF4B alone only marginally induced Plxna2 and Dcx activity, whereas SOX11 alone readily induced considerable Dcx and Plxna2 reporter activity. No change in activity over SOX11 alone was seen when TCF4A was co-expressed with SOX11 (Fig. 5F). In contrast, TCF4B co-expression strongly potentiated SOX11 induced reporter activities (Fig. 5F), indicating that TCF4B and SOX11 can cooperatively regulate the expression of target genes and supporting the general notion that Sox factors cooperate with other TFs to strongly induce target gene expression (Kamachi et al., 2000, Reiprich and Wegner, 2015).

8. It is clear unclear whether the authors think that Dcx and Plxna2 are contributing significantly to the TCF4 mutant phenotype. Radial migration does not seem to be affected which would be expected if Dcx is affected in the mutants. The authors should discuss and comment on this.

Answer: We would like to point out that previous studies have described a role for DCX in growth cone regulation and axonal growth e.g. (Yap et al., 2016, Bott et al., 2020). It is also of note that the DCX KO mouse model does not show a radial migration phenotype (Corbo et al., 2002), suggesting that mice can compensate for decreased DCX dosage in radial migration. We are alerting the reader to the fact that DCX has been suggested in the regulation of axonal development. The respective section reads as follows:
Line 326-334: We identified two potential common target genes of TCF4 and SOX11, Dcx and Plxna2, which have been shown to play important roles in axonal development and axon guidance (Rohm et al., 2000, Mitsogiannis et al., 2017, Deuel et al., 2006, Pilz et al., 1998, Yap et al., 2016, Bott et al., 2020) and are candidates to contribute to the observed dysgenesis of the forebrain commissures in Tcf4KO mice. It is, however, very likely that these two genes are only two examples out of a larger network of factors involved in commissural formation, which are dysregulate due to the changes in gene regulatory network activities caused by the loss of TCF4B.

9 & 10.The authors see an increase in SATB2 at the expense of CTIP2 expressing neurons. CTIP2 and SATB2 are known to be in a cross-regulatory network and repress each others expression during fate acquisition. The authors focus on SATB2 expressing cells and show that some of these are actually misplaced in the deeper cortical layers where CTIP2 neurons are expected. Does TCF4 regulate CTIP2 expression? Are the authors sure they are not comparing a mixed population of neurons in the mutant with SATB2 neurons in the control. If CTIP is

© 2021. Published by The Company of Biologists under the terms of the Creative Commons Attribution License (https://creativecommons.org/licenses/by/4.0/).

downregulated in the mutant neurons it is to be expected that SATB2 is not repressed and thus deep layer neurons are retaining SATB2 expression? This would confuse the scRNA- seq analysis. The authors should not use small numbers of marker genes and RNA expression to define cell types.
The authors should also compare the CTIP2 neuron populations in the mutant and the controls and extend the marker panel to examine whether they are including CTIP2 neurons in their SATB2 mutant populations.

Answer: We appreciate the concern of the reviewer that we may have compared mixed cell populations. We have now performed the single-cell analyses using the separate clusters for the upper layers and deep layers that were identified after clustering using SEURAT. The upper layer cluster consists mainly of intercortical projection neurons and is defined by the expression of various markers, e.g. Pou3f2(Brn2), Pou3f3 (Brn1), Cux1, Dok5, Bhlhe22 (Bhlhb5), Mef2c, Satb2. The deep layer cluster is defined by the expression of, e.g. Bcl11b(Ctip2), Fezf2, Tbr1, Sox5, Tle4 (see Figure 3A). These analyses showed similar results for the Satb2 cluster and the upper layer cluster indicating the validity of our analysis of the Satb2 cluster as both clusters should be mainly made up from intercortical projection neurons. For the deep layer cluster containing Ctip2 expressing cells considerable differences were found when compared to the Satb2 and upper layer cluster and gene regulatory networks for deep layer development were dysregulated (Figure S4, S5 and Supplemental Table 3,4 and 5).
The data is now integrated in the main text as follows:

Line 138-149: To ascertain that we did not introduce a bias in the analysis by defining the examined cluster only by Satb2 expression, which appears to be affected by Tcf4 knockout, we also analysed the dataset using the upper (UL) and deep layer (DL) cluster that had been assigned based on known marker expression (see Figs 3A and B). The UL cluster, which is mostly consisting of IPNs, was highly comparable to the Satb2 cluster. with 184 differentially regulated genes shared between the two cluster (Satb2 DEG 222; UL DEG 206; Overlap 184; Figs S4 A-D and Table S3). Moreover, GO terms were highly corresponding (Table S3). For the DL cluster similar GO terms were identified, however, the underlying DEGs diverged considerably from the Satb2 cluster (Satb2 DEG 222; DL DEG 165; Overlap 111; Figs S4 D-H, Table S4). These results support the validity of our analysis using the Satb2 cluster to examine Tcf4- dependent genetic programs in IPN development.

Line 162-169: As with the DEG analysis, GRN analysis was also performed with the limited Satb2, UL and DL datasets. In the limited Satb2 (Table S5) and the UL dataset we found - similar to our analyses of the full Satb2 dataset - IPN specific TF-dependent regulons such as Pou3f3 and Cux1 being differentially active (Figs S5 A-F and Table S5). In contrast, differentially active regulons in the DL cluster represented known TF for deep layer development, e.g. Sox5 or Foxp2 (Figs S5 G-L and Table S5) Further analysis was therefore done with the original Satb2 dataset.

Minor comments

11. What is the variance covered by tSNE1 and tSNE2 in the analyses?

Answer: Unfortunately, we cannot answer this question. The use of a tSNE-Plot does not allow to trace back the variance due to the use of non-linear dimensional reduction that does not conserve global structure.

12. It is surprising that the authors identify a significant number of GE-derived neurons in their sequencing. Is this due to contamination, the result of GE derived interneurons migrating dorsally, or a miss annotation? The authors should comment.

Answer: We thank the author for the comment. We performed sequencing analyses from cortical tissue at E18.5. At this time-point GE derived interneurons have already migrated into the cortical plate and are commonly found there. In addition, contamination during the preparation with neurons from subcortical regions cannot be excluded.

13. The coIP data are not convincing. The blots are not of good enough quality particularly for the overexpression Ips in Figure 3G. The lanes are all different sizes due to running and

© 2021. Published by The Company of Biologists under the terms of the Creative Commons Attribution License (https://creativecommons.org/licenses/by/4.0/).

buffer artefacts. The authors should also include in the legend of Figure 3G and 3H that ms and rb refer to the species in which the primary antibody was raised.

Answer: We have performed additional Co-IP experiments, which confirmed the previous data. We have included better quality blots in the new manuscript. As suggested, we are now stating the meaning of ms and rb in the Figure legends.

Reviewer 2 Comments for the Author:
Although the transcriptomics is informative, the analysis of the mutant phenotypes is superficial and the images of the stained sections are very poor (Figs 1C,H and 4A). It is not clear what histochemical method was used to stain the sections in Fig 1C and the commissures are barely visible in the wildtype cortices (Figs 1C, 4A). The sections used for the wildtype and mutant appear to be poorly matched (Figs 1C,D,H and 4A), making it difficult to directly compare sections. This will directly impact the results when quantify neuronal subtypes. The cortical thickness of the mutant (Fig. 1C and D) appears to be reduced in the mutant, however, this is not taken into account when quantifying the neuronal subpopulations. Note that Mesman et al (2020) report that TCF4 mutants exhibited a reduced cortical plate. We are not given any information as to how the quantification (Fig 1E) was performed and normalized.

Answer: As the commissural phenotype in TCF4 KO mice was previously described, we focused on the molecular analysis of the TCF4-dependent regulatory network in interhemispheric projection neurons and did not conduct more detailed histological analyses. As suggested by this reviewer we have added better quality pictures and higher magnification images to illustrate the commissural phenotype. Moreover, we have added a description of the histochemical method to Fig 1C.
We would like to mention that TCF4 knockout affects major brain structures such as the hippocampus making it difficult to perfectly match sections. We therefore included more representative images for the comparison of the genotypes along the rostral caudal axis (see Supplemental Figures 1 and 2).
We apologize for not stating clearly how we quantified the neuronal subpopulations and corrected this error in the revised manuscript. We reanalyzed the numbers of the neuronal subpopulation by taking the cortical thickness into account and state the numbers now as the density of marker positive cells per mm^2 of cortical area counted.

In Fig.1D the mutant cortices appear smaller and quite distorted. A thorough description of the cortical cytoarchitecture is lacking, making it difficult to interpret the phenotype. DiI tracing indicates that CC axons are delayed in crossing the midline. This could be due to the aberrant cortical architecture, ie. a non-cell autonomous effect. Thus, it is possible that axonal growth is only delayed or inhibited due to the local environment? This possibility should be investigated, as if true, the role of TCF4 would need to be revaluated. An in vitro assay would address this question.

Answer: We agree that it would be beneficial to extend the investigation on the non-cell autonomous and cell autonomous effect of TCF4. As suggested, we performed in vitro analyses and examined axonal outgrowth in primary neuron cultures of WT and KO neurons derived from the cortex on E16.5. We were able to show that TCF4 KO decreased length of the longest neurite, strongly indicating a cell autonomous effect of TCF4 KO on neurite growth. The data is presented in Figure 2E. The respective section reads as follows:
Line 103-107: Analysis of primary cortical neurons isolated from E16.5 embryos showed that the length of the longest neurite, which will usually be specified into the axon, was significantly reduced in Tcf4KO neurons in comparison with WT neurons (Fig. 2E). This observation suggested that disruption of a TCF4- dependent developmental program in neurons contributed to commissural agenesis in the present Tcf4KO mice.
We would also like to point out that we do not exclude non-cell autonomous effects but discuss the possibility that non-cell autonomous effects contribute to the commissural phenotype in Tcf4KO.
See line 299-304: As discussed, we could not corroborate the notion of Mesman et al. (2020) that Tcf4KO impairs midline formation, but it is important to note that our histological analyses cannot exclude subtle defects in midline development and axon guidance cues. Our finding of defective neurite outgrowth in isolated Tcf4KO cortical neurons, however, indicated that Tcf4KO directly

© 2021. Published by The Company of Biologists under the terms of the Creative Commons Attribution License (https://creativecommons.org/licenses/by/4.0/).

perturbs neurodevelopmental programs in cortical neurons.

The phenotype of the TCF4 mutants as described here appears to be distinct from that reported by Mesman et al (2020). In contrast to this manuscript, Mesman et al report an absence of midline remodelling and an increase in Ctip2+/Satb2+ neurons in layers V/VI. This should be discussed in the Discussion section. However, these differences may be resolved if a more thorough analysis is performed.

Answer: We thank the author for the comment and extended the discussion section accordingly. See line 273-281: The disparity regarding the midline formation between our study and the study by Mesman and colleagues may in principle be caused by the different Tcf4 knockout strategies and the remaining expression of shorter isoforms with DNA-binding potential. It is, however, important to keep in mind that the midline was analysed at different developmental time-points: while we performed our analyses on E16.5 – the time-point when the interhemispheric fissure is fusing, acute midline remodelling is occurring and pioneer axons are crossing into the contralateral hemisphere; Mesman and colleagues analysed the midline at P0, when interhemispheric fissure remodelling is mostly completed (Gobius et al., 2016).

Analysis of the axon tracts of the compound TCF4/Sox11 mutants is performed at p56 only. How does the phenotype of these mutants compare with the TCF4 mutants embryonically?

Answer: We understand the request as a question regarding if the axonal tracts are not developing normally or if they degenerate over time. Comparing the corpus callosum development embryonically will most likely lead to no conclusive answer as corpus callosum development spans into postnatal development. Especially caudal parts are established postnatally. We therefore chose to examine the axonal tracts at P7 when most developmental processes have taken place and developmental defects in the caudal part of the corpus callosum should be visible (See Figure S8). These analyses corroborated our findings from P56 indicating that the loss of the caudal corpus callosum is a developmentally cause phenotype.

Line 220-226: We also performed analyses at P7, i.e., around the time when all commissural axons have just crossed the midline and started innervating the contralateral hemispheres, in order to distinguish whether the commissural phenotype was caused by maldevelopment or by degeneration of IPNs or their projections. Again, we found a severely truncated CC and loss of the AC in double haploinsufficient mice (Fig. S8) strongly suggesting that the combined decrease in Sox11 and TCF4 gene dosage impaired commissural system development.

There is no description of the single cell FACS sorting protocol in the Materials and Methods.

Answer: Thank you for this comment. We apologize for the misunderstanding, but we did not perform single cell FACs sorting but used the whole single cell suspension of one cortex for the single cell experiment.

Were 3 independent biological replicates performed for the luciferase assays?

Answer: Thank you for this comment and apologize if that was not clearly stated. We performed three independent biological replicates and have now stated so in the Material and Methods section.

The proximity ligation assays have not been controlled appropriately. Irrelevant isotype-matched control antibodies should be paired with the specific antibodies.

Answer: Thank you for this comment. We controlled the proximity ligation assays as suggested. The controls indicate that the signal detected in the proximity ligation assays is specific. The data for the control experiments are presented in Supplemental Figure 6.

Some references are incomplete.

Answer: Thank you for this comment. We checked and completed the references.

© 2021. Published by The Company of Biologists under the terms of the Creative Commons Attribution License (https://creativecommons.org/licenses/by/4.0/).

References
BOTT, C. J., MCMAHON, L. P., KEIL, J. M., YAP, C. C., KWAN, K. Y. & WINCKLER, B. 2020. Nestin Selectively Facilitates the Phosphorylation of the Lissencephaly-Linked Protein Doublecortin (DCX) by cdk5/p35 to Regulate Growth Cone Morphology and Sema3a Sensitivity in Developing Neurons. *J Neurosci,* 40, 3720-3740.
CORBO, J. C., DEUEL, T. A., LONG, J. M., LAPORTE, P., TSAI, E., WYNSHAW-BORIS, A. & WALSH, C. A. 2002. Doublecortin is required in mice for lamination of the hippocampus but not the neocortex. *J Neurosci,* 22, 7548-57.
GOBIUS, I., MORCOM, L., SUAREZ, R., BUNT, J., BUKSHPUN, P., REARDON, W., DOBYNS, W. B., RUBENSTEIN, J. L., BARKOVICH, A. J., SHERR, E. H. & RICHARDS, L. J. 2016. Astroglial-Mediated Remodeling of the Interhemispheric Midline Is Required for the Formation of the Corpus Callosum. *Cell Rep,* 17, 735-747.
KAMACHI, Y., UCHIKAWA, M. & KONDOH, H. 2000. Pairing SOX off: with partners in the regulation of embryonic development. *Trends Genet,* 16, 182-7.
MESMAN, S., BAKKER, R. & SMIDT, M. P. 2020. Tcf4 encodes correct brain development during embryogenesis. *Mol Cell Neurosci,* 103502.
REIPRICH, S. & WEGNER, M. 2015. From CNS stem cells to neurons and glia: Sox for everyone. *Cell Tissue Res,* 359, 111-24.
YAP, C. C., DIGILIO, L., MCMAHON, L., ROSZKOWSKA, M., BOTT, C. J., KRUCZEK, K. & WINCKLER, B. 2016. Different Doublecortin (DCX) Patient Alleles Show Distinct Phenotypes in Cultured Neurons: EVIDENCE FOR DIVERGENT LOSS-OF-FUNCTION AND "OFF-PATHWAY" CELLULAR MECHANISMS. *J Biol Chem,* 291, 26613-26626.

Second decision letter

MS ID#: DEVELOP/2020/196022

MS TITLE: scRNA-Sequencing uncovers a TCF4-dependent transcription factor network regulating commissure development

AUTHORS: Marie-Theres Wittmann, Sayako Katada, Elisabeth Sock, Philipp Kirchner, Arif B Ekici, Michael Wegner, Kinichi Nakashima, Dieter Chichung Lie, and André Reis

I have now received all the referees reports on the above manuscript, and have reached a decision. The referees' comments are appended below, or you can access them online: please go to BenchPress and click on the 'Manuscripts with Decisions' queue in the Author Area.

The overall evaluation is positive and we would like to publish a revised manuscript in Development, provided that the referees' comments can be satisfactorily addressed. Please attend to all of the reviewers' comments in your revised manuscript and detail them in your point-by-point response. If you do not agree with any of their criticisms or suggestions explain clearly why this is so.

We are aware that you may currently be unable to access the lab to undertake experimental revisions. If it would be helpful, we encourage you to contact us to discuss your revision in greater detail. Please send us a point-by-point response indicating where you are able to address concerns raised (either experimentally or by changes to the text) and where you will not be able to do so within the normal timeframe of a revision. We will then provide further guidance. Please also note that we are happy to extend revision timeframes as necessary.

Reviewer 1

Advance summary and potential significance to field

In their manuscript Wittmann et al. addressed the function of TCF4 during cortical development. They confirm recent findings that deletion of the long variant of TCF is lethal and results in cortical

© 2021. Published by The Company of Biologists under the terms of the Creative Commons Attribution License (https://creativecommons.org/licenses/by/4.0/).

agenesis. They show that one effect of TCF4 mutation is an increase in SATB2 expression upper layer cortical neurons at the expense of CTIP2 expressing neurons. They show that although SATB2 expressing putative commissural neurons are generated in excess, the TCF mutant mice show an agenesis of most cortical commissural tracts.
They then performed scRNA-seq in order to try to identify the defect with a major focus on the SATB2 expressing neurons in the mutant compared to control. They identify transcriptional networks and transcription factor regulons that are miss-regulated in the TCF4 mutant neurons. They show TCF4-Sox11 cross-interactions and identify putative regulatory elements that, at least in heterologous cell systems, show a synergistic regulation by TCF4 and Sox11.

Comments for the author

In their revised manuscript the authors have addressed most of my concerns. However, I still have some issues that should be addressed to make the data more convincing.

Main concerns
1. The images in Figure 1D and Figure S2 are exactly the same. The image quality is still too low and there needs to be higher magnification images with cellular resolution. The authors should annotate the images to guide the non-expert reader to the important changes in the mutant brains. This is particularly important as the mutant brains are structurally very different to those of the controls.

2. The images in Figure 2B and C should also be better annotated.

3. Figure 2 E. There are 5 points on the bar graphs and an n=5 is stated. Presumably each point is the mean of individual cultures from single mice? Presumably, the authors counted many neurons from each mouse? This information should be provided and/or the individual measurements plotted on the graphs.
The quantification method should also be described in more detail in the Methods section. The cultures were prepared from the cortex of control and mutant mice but the authors need to focus this analysis on commissural neurons to be comparing like-with-like.

4. The authors still claim that the TCF4A does not interact with Sox11 by showing the co-IP data and detection of the longer form of TCF4. However, this co-IP should also be performed in the TCF4B KO cells.

5. It is unfortunate that the authors cannot show an in vivo association of Sox11 and Tcf4 with the endogenous promoters of Dcx and Plxna2. I feel this is a critical point that should be addressed better.
EMSA assays just show that a factor is able to bind a short piece of naked DNA that contains a consensus site, it is not proof that this site is bound in vivo at a relevant point in the cells lineage.

<u>Reviewer 2</u>

Advance summary and potential significance to field

This manuscript identifies novel binding partners for the TCF4 transcriptional regulator derived from gene regulatory network analysis based on single cell transcriptomics on loss-of-function mice. The described pipeline appears to yield verifiable candidate interactors as demonstrated by the investigation of TCF4-Sox11 interactions using co-immunoprecipitations and luciferase assays. However, I would note that although the compound TCF4/Sox11 mutant exhibits a similar phenotype, this does not offer conclusive evidence that TCF4 and Sox11 directly interact in vivo. Instead, it only indicates that these proteins act in the same pathway. The network analysis has identified a panel of other potential TCF4 cofactors, thereby providing the starting point for further exploration of TCF4 function.

Comments for the author

The authors have done a thorough job in addressing my comments. I have no further concerns.

© 2021. Published by The Company of Biologists under the terms of the Creative Commons Attribution License (https://creativecommons.org/licenses/by/4.0/).

Second revision

Author response to reviewers' comments

Response to Comments:
Reviewer 1 Comments for the author

In their revised manuscript the authors have addressed most of my concerns. However, I still have some issues that should be addressed to make the data more convincing.

Answer: We are happy to hear that we addressed most concerns of the reviewer.

Main concerns

1.The images in Figure 1D and Figure S2 are exactly the same. The image quality is still too low and there needs to be higher magnification images with cellular resolution. The authors should annotate the images to guide the non-expert reader to the important changes in the mutant brains. This is particularly important as the mutant brains are structurally very different to those of the controls.

Answer: Compared to Figure 1D, Figure S2 contained one additional panel of images illustrating the loss of the corpus callosum in TCF4 KO mice at a rostral level. We, however, agree with reviewer 1 that Figure S2 does not add substantial information and have therefore removed Figure S2 for this revision. We have to apologize for the low quality of the higher magnification images, we had to condense the images to abide by the data maxima of Development which resulted in decreased resolution. With the original resolution readers will be indeed able to zoom into the images and view the images with cellular resolution. We will contact the editorial office for guidance on how to handle this problem. We have added the requested annotations to the images.

2.The images in Figure 2B and C should also be better annotated.

Answer: We have added the requested annotations to the images.

3.Figure 2 E. There are 5 points on the bar graphs and an n=5 is stated. Presumably each point is the mean of individual cultures from single mice? Presumably, the authors counted many neurons from each mouse? This information should be provided and/or the individual measurements plotted on the graphs. The quantification method should also be described in more detail in the Methods section. The cultures were prepared from the cortex of control and mutant mice but the authors need to focus this analysis on commissural neurons to be comparing like-with-like.
Answer: Thank you for this suggestion. We have repeated the experiment and stained for SATB2 and CTIP2. We only analysed neurons that expressed only SATB2 and not CTIP2 to ensure that only commissural neurons are included in the analysis. We apologize for not clearly stating how the analysis was executed. We have added the information in the main text and figure legend and added a more detailed description in the Methods section.
Cells were fixed after 3 days in vitro and stained with rb TUBB3 (Abcam, 18207) 1:500, ms SATB2 (Santa Cruz, sc-81376) 1:500, rt CTIP2 (Abcam, 18465) 1:500 and DAPI. Single neurons, which did not contact other neurons in the vicinity and only expressed SATB2, but not CTIP2, were imaged for analysis to ensure that only intercortical projection neurons are compared. 20 cells per animal were analysed. Longest neurite lengths were measured from the soma to the tip of the longest neurite using the Simple neurite tracer tool of Fiji (Longair et al., 2011).

4.The authors still claim that the TCF4A does not interact with Sox11 by showing the co-IP data and detection of the longer form of TCF4. However, this co-IP should also be performed in the TCF4B KO cells.

© 2021. Published by The Company of Biologists under the terms of the Creative Commons Attribution License (https://creativecommons.org/licenses/by/4.0/).

Answer: We thank the reviewer for this comment and have performed the experiment. See Figure S10 and lines 335-337:
We were also able to show that TCF4B does not impede TCF4A binding to SOX11 as TCF4A was not co-immunoprecipitated with SOX11 when using protein isolates from the neocortex of KO mice (Fig. S10C).

5.It is unfortunate that the authors cannot show an in vivo association of Sox11 and Tcf4 with the endogenous promoters of Dcx and Plxna2. I feel this is a critical point that should be addressed better. EMSA assays just show that a factor is able to bind a short piece of naked DNA that contains a consensus site, it is not proof that this site is bound in vivo at a relevant point in the cells lineage.

Answer: We agree that ChIP-PCR from tissue would add further evidence that Sox11 and Tcf4 associate in vivo with the promotors of Dcx and Plxna2. However, despite our best efforts, we found that currently commercially available antibodies were not suitable for such analyses and cannot conduct the requested experiment. We would like to point out that we provide 1) proximity ligation assay based evidence that Sox11 and Tcf4 interact in differentiating embryonic cortical neural precursor cultures, 2) biochemical evidence for in vivo interaction of Sox11 and Tcf4, 3) Luciferase reporter -based evidence for cooperativity of Sox11 and Tcf4 on candidate target genes, 4) EMSA assays based evidence that Tcf4 and Sox11 can bind to predicted consensus sites in common candidate target genes, and 5) genetic evidence for cooperativity of Tcf4 and Sox11. Given these evidences, we feel that it is justified to state that "…Collectively, these data support the hypothesis that TCF4 and SOX11 can cooperate to regulate transcription of common target genes…."(lines 249-50).

Reviewer 2 Comments for the author
The authors have done a thorough job in addressing my comments. I have no further concerns.

Answer: We thank the reviewer for his/her helpful comments.

Third decision letter

MS ID#: DEVELOP/2020/196022

MS TITLE: scRNA-Sequencing uncovers a TCF4-dependent transcription factor network regulating commissure development

AUTHORS: Marie-Theres Wittmann, Sayako Katada, Elisabeth Sock, Philipp Kirchner, Arif B Ekici, Michael Wegner, Kinichi Nakashima, Dieter Chichung Lie, and André Reis
ARTICLE TYPE: Research Article

I am happy to tell you that your manuscript has been accepted for publication in Development, pending our standard ethics checks.

© 2021. Published by The Company of Biologists under the terms of the Creative Commons Attribution License (https://creativecommons.org/licenses/by/4.0/).
